# Healthful and unhealthful plant-based diets and site-specific cancer risk: a systematic review and meta-analysis of observational studies

**DOI:** 10.1007/s00394-026-03948-2

**Published:** 2026-04-21

**Authors:** Mercedes Gil-Lespinard, Lucía Iglesias-Vázquez, Paula Jakszyn

**Affiliations:** 1 Unit of Nutrition and Cancer, Cancer Epidemiology Research Program, Catalan Institute of Oncology (ICO), L’Hospitalet de Llobregat, Spain; 2 Nutrition and Cancer Group, Epidemiology, Public Health, Cancer Prevention and Palliative Care Program, Bellvitge Biomedical Research Institute (IDIBELL), L’Hospitalet de Llobregat, Spain; 3 Departament de Bioquímica i Biotecnologia, Universitat Rovira i Virgili, Desenvolupament i Salut Mental ANUT-DSM, Alimentaciò, Nutrició, Reus, Spain; 4 Institut D’Investigació Sanitària Pere Virgili (IISPV), Reus, Spain; 5 Blanquerna School of Health Sciences, Ramon Llull University, Barcelona, Spain

**Keywords:** Plant-based diet, Cancer, Meta-analysis, Observational study, Epidemiology, Diet quality

## Abstract

**Purpose:**

Plant-based diets have been proposed as beneficial for cancer risk reduction, though their impact may depend on their nutritional quality. We conducted a systematic review and meta-analysis of observational studies to examine associations of overall Plant-Based Diet Index (PDI), Pro-Vegetarian Dietary Pattern (PVG), healthful PDI (hPDI), and unhealthful PDI (uPDI), with site-specific cancer risk.

**Methods:**

PubMed and Web of Science were searched (June 2018 – December 2025) for cohort and case-control studies evaluating these associations. Meta-analyses were conducted for cohort studies, when ≥ 3 studies assessed the same cancer site and outcome, and if dietary indices were measured on a comparable scale. Pooled hazard ratios were calculated using random-effects models. Leave-one-out sensitivity analyses were conducted to evaluate the influence of individual studies on the pooled estimates.

**Results:**

A total of 19 cohort studies and 13 case-control studies were reviewed. Meta-analysis showed that per 10-unit increase, overall and healthful plant-based dietary indices were associated with lower risks of breast cancer (8 and 6% lower risk, respectively), colorectal cancer (5% lower risk each), and liver cancer (17 and 23% lower risk, respectively). Higher uPDI was associated with 3% higher breast cancer risk. Evidence for other cancer sites was promising yet limited. Findings were generally consistent in direction, though some associations were moderately influenced by individual studies.

**Conclusion:**

Plant-based diets may reduce cancer risk, particularly when they prioritize healthful, minimally processed plant foods. Incorporating this distinction into clinical and policy recommendations may support practical, scalable strategies to reduce cancer burden.

**Supplementary Information:**

The online version contains supplementary material available at 10.1007/s00394-026-03948-2.

## Introduction

Cancer remains a leading cause of morbidity and mortality worldwide [1], with lifestyle and dietary factors playing a significant role in both its development and survival [2]. Plant-based diets have been associated with a reduced risk of various chronic diseases, including cardiovascular diseases and type 2 diabetes [3–7]. Earlier studies on plant-based diets and cancer risk often conceptualized plant-based eating dichotomously according to the presence or absence of animal foods [8]. However, from a public health perspective, an important question is whether gradually shifting toward diets richer in plant foods – not necessarily excluding animal-derived foods completely – is associated with better health outcomes, including lower cancer risk [7, 9]. Such an approach aligns more closely with real-world dietary behaviors and may offer greater potential for population-level impact. In this context, ultra-processed foods have become widespread in the diets of many populations, paralleling the rising incidence of non-communicable chronic diseases, including cancer [10]. This trend includes an increasing number of ultra-processed plant-based products which may adversely impact health due to their poor nutritional quality [7, 10, 11]. Grouping all plant-based diets together may obscure important differences in dietary quality, as plant foods vary widely in their nutritional composition and biological effects. To capture differences in diet quality within plant-based dietary patterns, several indices have been developed, including the Pro-Vegetarian Dietary Pattern (PVG), the overall Plant-Based Diet Index (PDI), the healthful PDI (hPDI), and the unhealthful PDI (uPDI) [3, 7]. The PVG emphasizes plant-derived foods while moderately allowing for animal products, assigning higher scores to greater plant and lower animal food consumption [3]. The PDI gives positive scores to all plant foods, regardless of nutritional quality, and negative scores to animal foods [7]. The hPDI assigns greater scores to health-promoting plant foods – such as fruits, vegetables, whole grains, nuts, and legumes – while penalizing both animal products and less nutritious plant foods [7]. Conversely, the uPDI prioritizes intake of lower-quality plant foods, including refined grains, starchy vegetables, sugar-sweetened beverages, sweets, and other ultra-processed plant-based products, while assigning lower scores to both healthful plant and animal foods [7]. Together, these indices enable a more differentiated evaluation of plant-based dietary patterns, addressing limitations of earlier studies that considered plant-based dietary patterns as a homogeneous exposure [12].

Recent large-scale prospective analyses have extended this line of research by examining plant-based dietary patterns in relation to multimorbidity and mortality risk [13, 14]. However, given the biological heterogeneity across cancer types and the increasing recognition that diet quality within plant-based patterns is critical, a comprehensive synthesis focused on site-specific cancer risk was warranted.

The aim of this systematic review and meta-analysis was to synthesize, based on findings from observational studies, the available evidence on the association between plant-based dietary indices – PVG, PDI, hPDI and uPDI – and site-specific cancer risk. In a case–control study included, the pro-vegetarian dietary pattern has also been further classified into healthful and unhealthful variants (hPVG and uPVG, respectively) based on the quality of plant foods consumed [15]; however, these distinctions have not been applied across cohorts and were therefore not the primary focus of this study.

## Methods

### Protocol and reporting standards

**Fig. 1:**
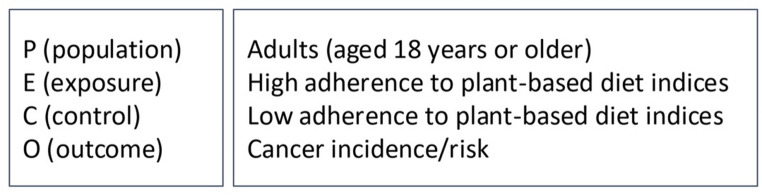
Population, exposure, control, outcomeand study design (PECO(s) guideline)

This systematic review was conducted in accordance with the Preferred Reporting Items for Systematic Reviews and Meta-Analyses (PRISMA) statement [16] as detailed in Supplementary Table 1. Reporting also aligns with the MOOSE recommendations for meta-analyses of observational studies [17]. The protocol of this review was registered in the PROSPERO International Prospective Register of Systematic Reviews (registration number: CRD42025639327). The PECO(s) guideline was used to guide the eligibility criteria and structure the research question (Fig. 1).

### Literature search and study selection

The literature search was conducted on PubMed and Web of Science. The search covered publications from 1st June 2018 to 16th December 2025 due to the relatively recent emergence of this exposure in the literature [3, 7]. The search strategy combined terms related to plant-based dietary indices and cancer outcomes, using the following combination: (vegetarian OR “plant-based diet” OR “plant-based diet index” OR “plant-based dietary pattern”) AND (cancer OR “breast cancer” OR “colorectal cancer” OR “digestive cancer” OR “stomach cancer” OR “liver cancer” OR “pancreatic cancer” OR “bile tract cancer” OR “prostate cancer” OR “lung cancer”) AND (“cancer risk” OR “cancer incidence”) AND (observational OR case-control OR cohort) NOT (review OR “systematic review” OR meta-analysis). Filters applied included studies performed in humans and English language. Manual screening of the reference lists of eligible articles was also performed to identify additional relevant studies. Two authors (MG-L and LI-V) independently screened the titles, abstracts, and full texts of retrieved records. Inclusion criteria were: (1) cohort or case-control design; (2) English language; (3) adult population; (4) availability of full text; (5) evaluation of the association between a plant-based diet index (PDI, hPDI, uPDI, PVG, hPVG, uPVG) and cancer risk/incidence. We restricted inclusion to cohort and case-control studies, given their suitability for evaluating long-term dietary exposures and cancer incidence in large, free-living populations. Comparability criteria were defined a priori based on alignment with original index construction, food group scoring direction, and analytical scale compatibility. Accordingly, only plant-based dietary indices constructed using published and widely accepted scoring methodologies were included. Eligible studies were required to derive indices from the original frameworks, preserve the directional scoring of healthful and unhealthful plant food groups, and apply negative or lower scoring to animal-derived foods. In addition, indices had to be reported on a continuous or ordinal scale that allowed comparison across exposure categories or transformation for quantitative synthesis. Any discrepancies were resolved through discussion with a third author until consensus was reached.

### Data extraction

From each eligible study, the following data were extracted: first author, year of publication, country, study name, cancer site, sample size, number of cases (in cohort), number of cases and controls (in case-control studies), study base (in case-control studies), duration of follow-up (in cohort) or study period (in case-control studies), participant characteristics (age and sex), dietary assessment method, type of plant-based diet index, adjustment variables, and the most fully adjusted hazard ratios (HRs) or odds ratios (ORs) with 95% confidence intervals.

### Quality assessment

The quality of the included cohort studies was assessed using the ROBINS-E (Risk of Bias in Nonrandomized Studies of Exposures) tool [18], which evaluates bias in the selection of participants, classification of exposures, measurement of outcomes, and control of confounding, as well as biases due to deviations from intended interventions and missing data. For case-control studies, the Newcastle-Ottawa Scale (NOS) [19] was used, which evaluates the adequacy of case definition, representativeness of cases, selection of controls, and definition of controls (Selection: up to 4 stars); control for confounding (Compatibility: up to 2 stars); ascertainment of exposure, consistency in measurement methods for cases and controls, and evaluation of the non-response rate (Exposure: up to 3 stars). Higher scores reflect greater methodological quality.

### Meta-analysis methods

Case-control studies were not pooled quantitively and were considered as complementary evidence, given their increased susceptibility to recall bias, selection bias, and reverse causality, the heterogeneity between population-based and hospital-based studies, and the greater instability of effect estimates [20]. Cohort studies were included in the meta-analysis if they reported on the same cancer site and outcome, and if their dietary indices were measured on a comparable scale. At least three eligible studies were required for a meta-analysis to be conducted. When studies reported results stratified by sex, each subgroup was treated as an independent analysis. For each study, we extracted the most fully adjusted risk estimates, which in cohorts with repeated dietary assessments were based on time-updated or cumulative average dietary exposures, as reported in the original publications. The main approach used was the standardization to a 10-unit increase in the diet index, allowing for comparability across studies. When needed, relative risks were recalculated per 10-unit increment using reported beta coefficients and standard deviations. For studies reporting only categorical results, category midpoints were used to estimate dose-response relationships using generalized least squares regression [21, 22]. Complementary, or when standardization was not possible, estimation of the effect comparing the highest versus the lowest category of diet index (e.g. Q5 vs. Q1, T3 vs. T1) was used. Random-effects models were applied to account for between-study heterogeneity. Study weighs were calculated based on the standard error of the log-transformed relative risk. The certainty of evidence for each cancer-specific outcome was assessed using the GRADE approach [23]. Certainty rating started at moderate. Evidence was downgraded based on risk of bias, inconsistency, and imprecision, when applicable. Publication bias was not formally assessed due to the small number of studies per outcome [24]. Certainty ratings were assigned separately for each dietary index and cancer site.

### Sensitivity analyses and heterogeneity

To assess the robustness of the meta-analytic estimates, leave-one-out sensitivity analyses were conducted by sequentially excluding each study from the analysis. Heterogeneity across studies was assessed using Cochran’s Q test and the I^2^ statistic [25]. All analyses were performed using the ‘meta’ [26] and ‘metafor’ [27] packages in Rstudio, R (version 2024.12.1) [28].

## Results

### Overview of included studies

**Fig. 2:**
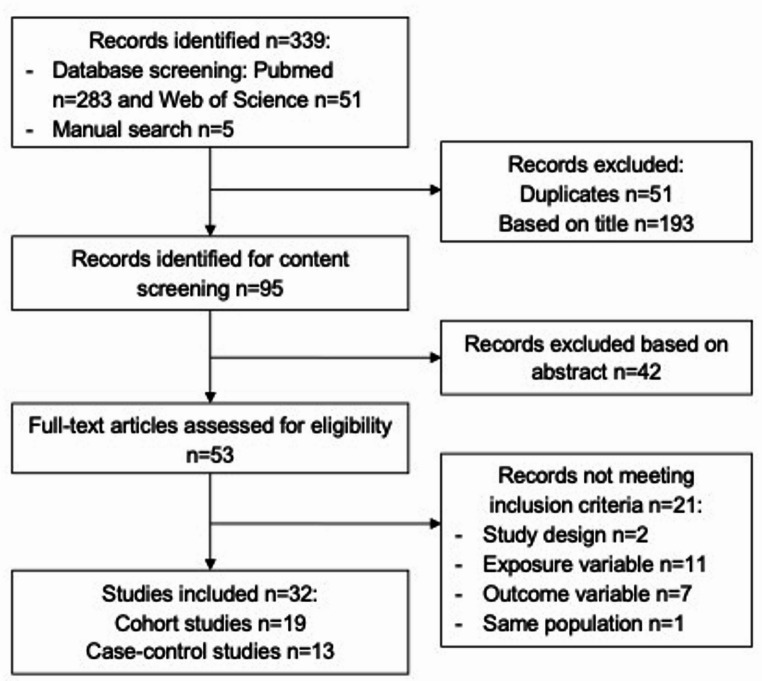
Flow diagram of literature search and selection process adapted from Preferred Reporting Items for Systematic Reviews and Meta-Analyses (PRISMA)

The literature search yielded 339 records (283 from PubMed, 51 from Web of Science, and 5 from reference screening). After removing duplicates (*n* = 51), 288 titles and abstracts were screened, of which 235 were excluded for irrelevance. Of the remaining 53 full-text articles, 21 were excluded due to inappropriate design (*n* = 2), lack of relevant exposure (*n* = 11), outcome not meeting the inclusion criteria (*n* = 7), or population overlap with another included study (*n* = 1). Thus, 32 observational studies (19 cohort and 13 case-control) were retained for analysis (Fig. 2**)**.

### Cohort studies

Table 1 shows a summary of the nineteen cohort studies included [29–46], primarily conducted in Europe (France, Italy, Spain, the United Kingdom) and the United States. More than one million participants were assessed (*n* = 1,209,947), with a mean age of participants and follow-up duration of 54.7 and 13.5 years, respectively. In total, these studies covered over 56,500 cancer cases, with breast, colorectal, and prostate cancer as the most frequently studied outcomes. Most of the studies used validated food frequency questionnaires (FFQs) as dietary assessment method, except two that used web-based 24-hour recalls. Dietary exposures were defined using standard scoring systems for PDI, hPDI, uPDI, PVG, hPVG, and uPVG, based on published methodologies [3, 7]. Covariate adjustment generally included age, sex (when not analyzed separately), body mass index, smoking status, educational attainment, physical activity, and total energy intake, as well as cancer-specific factors such as family history of cancer, hormone replacement therapy for breast cancer, recent endoscopic screening for colorectal cancer, hepatitis B or C infection for liver cancer, and other relevant cancer-specific covariates, as appropriate.

**Table 1:** Baseline characteristics of included cohort studies (*n* = 19)

References	Cohort name, region	Cancer site	Sample size	Follow-up (mean years)	Dietary index	Dietary assessment method	Exposure assessment timing
Kane-Diallo [29]	NutriNet Santé cohort, France	Breast, prostate, lung, digestive organs	42,544	4.3	PVG	Three validated web-based 24 HDRs	Baseline and every 6 months
Martínez et al. [31]	Moli-Sani Study, Italy	Breast, prostate, digestive organs, respiratory tract, genitourinary organs, lymphatic/ hematopoietic, brain/ nervous system	22,081	12.9	PDI, hPDI, and uPDI	Validated semi-quantitative EPIC FFQ	Baseline
Thompson AS et al. [47]	UK Biobank, UK	Breast, Colorectum, Prostate	126,394	range 10.6-12.2	hPDI and uPDI	Validated Oxford WebQ 24 HDR	Baseline
Romanos-Nanclares et al. [32]	SUN cohort, Spain	Breast	10,812	11.5	PDI, hPDI, and uPDI	Validated semi-quantitative 136-item FFQ	Baseline
Romanos-Nanclares et al. [33]	NHS and NHSII, USA	Breast	169,985	28.5	PDI, hPDI, and uPDI	Validated > 130-item FFQ	Baseline and every 4 years
Shah et al. [34]	E3N, France	Breast	65,574	total 21.0	hPDI and uPDI	Validated 208-item FFQ	Baseline and follow-up
Shah et al. [41]	EPIC, Europe	Breast	258,343	median 14.9	PDI, hPDI, and uPDI	Validated semi-quantitative EPIC FFQ	Baseline
Kim et al. [35]	Multiethnic Cohort Study, USA	Colorectum	173,427	total 19.2	PDI, hPDI, and uPDI	Validated > 180-item quantitative FFQ	Baseline
Kim et al. [30]	NHS, NHSII and HPFS, USA	Colorectum, pancreas, liver, oral cavity/ oropharynge, esophagus, small intestine, colon, rectum, accessory organ, biliary tract	211,673	23.2	PDI, hPDI, and uPDI	Validated > 130-item FFQ	Baseline and every 4 years
Liu et al. [36]	UK Biobank, UK	Colorectum	186,675	9.5	PDI, hPDI, and uPDI	Validated Oxford WebQ 24 HDR	Baseline
Yuan et al. [44]	SCCS, USA	Colorectum, liver	71,533	6.0	PDI, hPDI, and uPDI	Semi-quantitative 89-item FFQ	Baseline
Loeb et al. [37]	HPFS, USA	Prostate	47,239	20.7	PDI and hPDI	Validated semi-quantitative 152-item FFQ	Baseline and every 4 years
Wei et al. [40]	PLCO Cancer Screening Trial, USA	Lung	98,459	8.9	PDI, hPDI, and uPDI	Validated 124-item diet history questionnaire	Baseline
Zhu et al. [46]	UK Biobank, UK	Lung	189,541	range 8.0–9.0	PDI, hPDI, and uPDI	Validated Oxford WebQ 24 HDR	Baseline
Zhong et al. [38]	PLCO Cancer Screening Trial, USA	Pancreas	101,748	8.9	PDI, hPDI, and uPDI	Validated 124-item diet history questionnaire	Baseline
Thi-Hai Pham et al. [45]	SCHS, Singapore	Pancreas	63,275	17.6	PDI, hPDI, and uPDI	Validated 165-item FFQ	Baseline
Dong et al.[42]	UK Biobank, UK	Liver	187,781	14.0	PDI, hPDI, and uPDI	Validated Oxford WebQ 24 HDR	Baseline
Kim et al. [39]	Multiethnic Cohort Study, USA	Hepatocellular carcinoma	170,321	19.6	PDI, hPDI, and uPDI	Validated > 180-item quantitative FFQ	Baseline
Pham et al. [43]	SCHS, Singapore	Hepatocellular carcinoma	63,275	17.6	PDI, hPDI, and uPDI	Validated 165-item FFQ	Baseline

FFQ: food frequency questionnaire, 24-HDR: 24-hour dietary recall, HR: hazard ratio, UK: United Kingdom; USA: United States of America. *Study names*: E3N: Étude Épidémiologique auprès de femmes de la Mutuelle Générale de l’Éducation Nationale, EPIC: European Prospective Investigation into Cancer and Nutrition, HPFS: Health Professionals Follow-up Study, NHS: Nurses’ Health Study, PLCO: Prostate, Lung, Colorectal, and Ovarian Cancer Screening Trial, SCCS: Southern Community Cohort Study; SCHS: Singapore Chinese Health Study; SUN: Seguimiento Universidad de Navarra. *Dietary indices*: PDI: plant-based diet index, hPDI: healthful plant-based diet index, uPDI: unhealthful plant-based diet index, PVG: pro-vegetarian pattern.

### Case-control studies

Thirteen case-control studies were included [15, 48–59], predominantly hospital-based (matching by age, sex and often also by recruitment center or socioeconomic status), and conducted in Asia (Iran and China) and Europe (Spain and Italy). Participant age ranged from 19 to 80 years, and more than 13,500 cancer cases were analyzed. Breast and colorectal cancer were the most frequent outcomes. Most case-control studies used validated FFQs and applied comparable PDI scoring methods. Covariate adjustment was generally comprehensive, including age, sex (when not analyzed separately), BMI, smoking status, education, physical activity, and total energy intake. Supplementary Table 2 shows a summary of the results reported by the case-control studies included.

### Risk of bias and certainty of the evidence

Overall, the risk of bias of the included cohort studies was judged as moderate (Supplementary Fig. 1). Bias due to confounding (Domain 1) was rated as presenting some concerns across studies, reflecting the observational design and potential for residual confounding. Bias related to exposure measurement was generally low; however, some concerns were identified in studies using a single 24-hour dietary recall at baseline (UK Biobank [36, 42, 46] and SCCS [44]) and in the EPIC cohort [41] due to heterogeneity in dietary assessment methods across centers. Bias in participant selection (Domain 3) was generally low, with some concerns noted for selected cohorts with specific recruitment frameworks or highly selected populations [32, 37, 38, 40]. Bias due to post-exposure interventions (Domain 4) was mostly rated as presenting some concerns, except for cohorts with repeated dietary assessments [29, 30, 33, 34, 37], which were judged at low risk. Bias due to missing data, outcome measurement, and selective reporting (Domains 5–7) was consistently rated as low. For case-control studies, the NOS scores ranged from seven to eight, indicating good methodological quality (Supplementary Table 3). While the majority adequately defined cases and controls and applied appropriate dietary assessment methods, several had potential limitations related to selection bias (e.g., hospital-based controls), recall bias due to retrospective dietary reporting, and incomplete information on response rates. Nevertheless, most case-control studies adjusted for key confounders and used consistent methodologies for cases and controls.

Overall certainty of evidence ranged from moderate to very low across cancer sites, mainly reflecting differences in heterogeneity, precision, and number of contributing studies for each meta-analysis (Supplementary Table 4).

### Breast cancer

Breast cancer risk was evaluated in seven cohort studies conducted in Europe and the United States [29, 31–34, 41, 47]. Overall, most cohort studies reported inverse associations for overall and hPDI, whereas associations with the uPDI were generally positive or null (Supplementary Table 5).

**Fig. 3:**
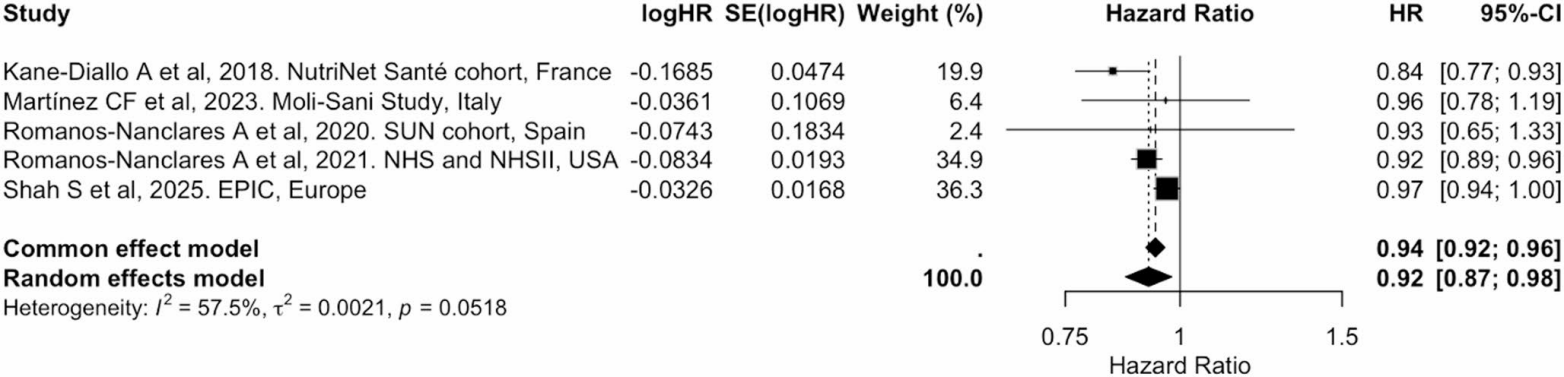
Meta-analysis of cohort studies assessing plant-based/pro-vegetarian dietary index (PDI/PVG) and breast cancer risk: forest plot showing pooled hazard ratios for 10-unit increase in index

**Fig. 4:**
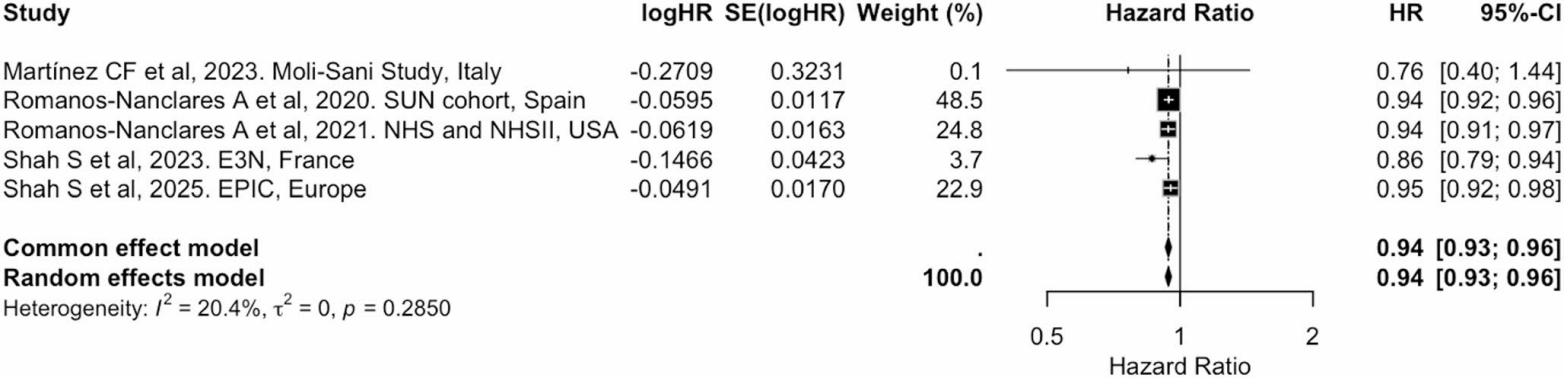
Meta-analysis of cohort studies assessing healthful plant-based dietary index (hPDI) and breast cancer risk: forest plot showing pooled hazard ratios for 10-unit increase in index

**Fig. 5:**
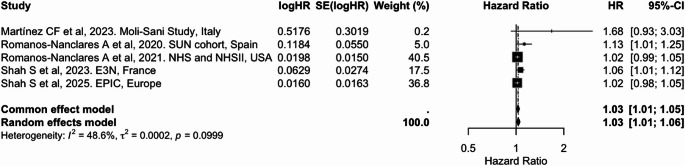
Meta-analysis of cohort studies assessing unhealthful plant-based dietary index (uPDI) and breast cancer risk: forest plot showing pooled hazard ratios for 10-unit increase in index

In the meta-analyses, higher adherence to overall plant-based dietary patterns (PDI/PVG) was associated with an 8% lower risk of breast cancer per 10-unit increase (pooled HR = 0.92; 95% CI: 0.87, 0.98), with moderate heterogeneity (I² = 57.5%) (Table 2 and Fig. 3). Similarly, higher adherence to an hPDI was consistently associated with a 6% lower risk [0.94 (0.93, 0.96); I² = 20.4%] (Table 2 and Fig. 4). In contrast, adherence to an uPDI was associated with a modest but statistically significant 3% increased risk of breast cancer [1.03 (1.01, 1.06)], with moderate heterogeneity (I² = 48.6%) (Table 2 and Fig. 5). These findings were broadly consistent with categorical exposure contrasts (Supplementary Figs. 2 to 4).

Evidence from case–control studies, all conducted in Iran [48, 53–55, 59], was generally directionally consistent with the cohort findings but showed larger and more heterogeneous effect estimates (Supplementary Table 2). Most case–control studies reported inverse associations between hPDI and breast cancer risk and positive associations for uPDI, while associations for overall PDI were less consistent. Given the hospital-based design of most case–control studies and their greater susceptibility to recall and selection bias, these results were considered supportive but were not quantitatively pooled.

Overall, evidence from prospective cohort studies supports a protective association of healthful plant-based dietary patterns and a detrimental association of unhealthy plant-based dietary patterns with breast cancer risk, with case–control studies providing complementary but less robust evidence.

**Table 2:** Summary of pooled associations between plant-based dietary indices and cancer risk

Cancer site	Dietary index	Exposure contrast	Included studies (k)	Cancer cases (*n*)	Pooled HR (95% CI)	I² (%)
Breast	PDI/PVG	Per 10-unit increase	5	19,898	0.92 (0.87, 0.98)	57.5
Breast	hPDI	Per 10-unit increase	5	23,379	0.94 (0.93, 0.96)	20.4
Breast	uPDI	Per 10-unit increase	5	23,379	1.03 (1.01, 1.06)	48.6
Colorectum	PDI	Per 10-unit increase	5	11.716	0.95 (0.91, 0.98)	66.7
Colorectum	hPDI	Per 10-unit increase	5	11.716	0.95 (0.92, 0.98)	55.2
Colorectum	uPDI	Per 10-unit increase	5	11.716	1.03 (0.99, 1.06)	63.1
Prostate	PDI/PVG	Per 10-unit increase	3	7,117	0.95 (0.72, 1.24)	96.7
Liver	PDI	Per 10-unit increase	4	1,390	0.83 (0.71, 0.97)	0.0
Liver	hPDI	Per 10-unit increase	4	1,390	0.77 (0.66, 0.90)	0.0
Liver	uPDI	Per 10-unit increase	4	1,390	1.06 (0.92, 1.22)	0.0
Lung	PDI/PVG	Highest vs. lowest category	3	2,731	0.76 (0.68, 0.85)	20.9

CI: confidence interval, HR: hazard ratio. *Dietary indices*: PDI: plant-based diet index, hPDI: healthful plant-based diet index, uPDI: unhealthful plant-based diet index, PVG: pro-vegetarian pattern. For comparability across studies, pooled estimates are presented per 10-unit increase in the dietary index when data allowed. For lung cancer, meta-analysis was conducted using the highest versus lowest category contrast because insufficient data were available to standardize estimates to a per 10-unit increase. PDI and PVG were considered equivalent exposures due to their comparable construct and scoring [3, 7]. I² represents the percentage of total variation across studies attributable to between-study heterogeneity; values > 50% indicate substantial heterogeneity.

CI: confidence interval, HR: hazard ratio, SE: standard error. I² represents the percentage of total variation across studies attributable to between-study heterogeneity; values > 50% indicate substantial heterogeneity. τ² represents the estimate of between-study variance in the random-effects model. PDI and PVG were considered equivalent exposures due to their comparable construct and scoring.

CI: confidence interval, HR: hazard ratio, SE: standard error. I² represents the percentage of total variation across studies attributable to between-study heterogeneity; values > 50% indicate substantial heterogeneity. τ² represents the estimate of between-study variance in the random-effects model.

CI: confidence interval, HR: hazard ratio, SE: standard error. I² represents the percentage of total variation across studies attributable to between-study heterogeneity; values > 50% indicate substantial heterogeneity. τ² represents the estimate of between-study variance in the random-effects model.

### Colorectal cancer

Five cohort studies evaluated the association between plant-based dietary indices and colorectal cancer risk (Table 1), including large prospective cohorts from the United States and the United Kingdom [30, 35, 36, 44]. Data from the Multiethnic Cohort Study [35] was reported separately by sex, and was therefore analyzed as two separate studies. In individual cohort analyses, higher adherence to overall PDI and hPDI was generally associated with a lower risk of colorectal cancer, whereas uPDI tended to show null or positive associations (Supplementary Table 5).

**Fig. 6:**
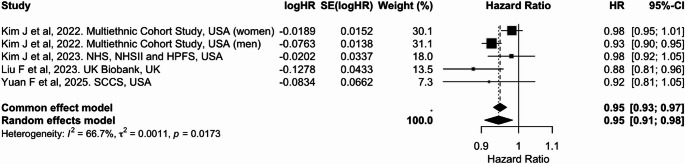
Meta-analysis of cohort studies assessing plant-based dietary index (PDI) and colorectal cancer risk: forest plot showing pooled hazard ratios for 10-unit increase in index

**Fig. 7:**
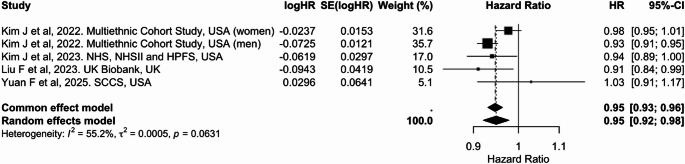
Meta-analysis of cohort studies assessing healthful plant-based dietary index (hPDI) and colorectal cancer risk: forest plot showing pooled hazard ratios for 10-unit increase in index

**Fig. 8:**
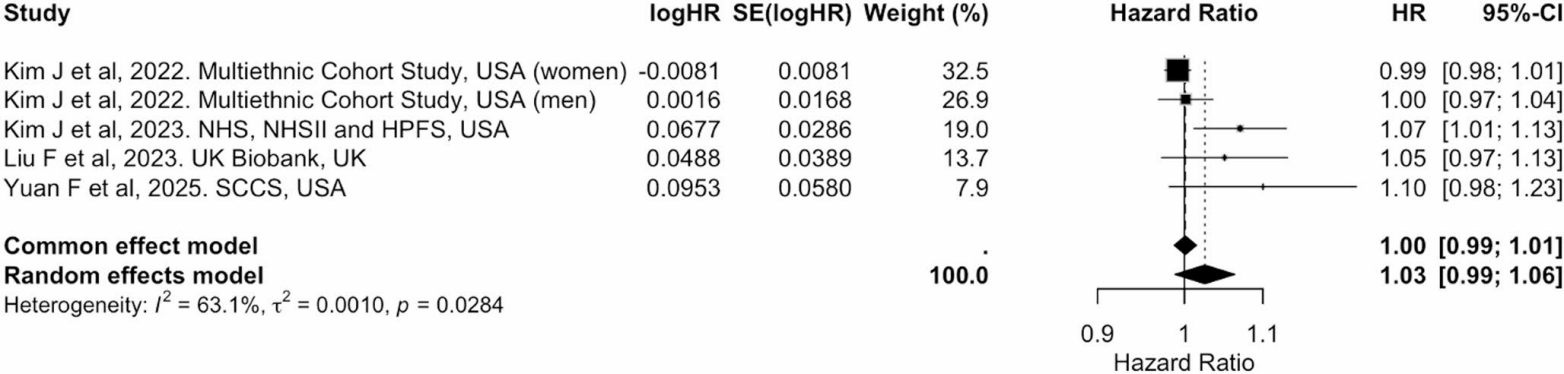
Meta-analysis of cohort studies assessing unhealthful plant-based dietary index (uPDI) and colorectal cancer risk: forest plot showing pooled hazard ratios for 10-unit increase in index

In the meta-analysis, five studies were included for continuous comparisons standardized to a 10-unit increase in the dietary index (Table 2). Each 10-unit increase in PDI was associated with a 5% lower risk of colorectal cancer [pooled HR: 0.95 (95% CI: 0.91, 0.98)], with substantial heterogeneity (I² = 66.7%) (Fig. 6). Similarly, higher hPDI was associated with a 5% reduced risk [0.95 (0.92, 0.98), I² = 55.2%] (Fig. 7). In contrast, no significant association was observed for uPDI [1.03 (0.99, 1.06)], although heterogeneity was considerable (I² = 63.1%) (Fig. 8). Overall, hPDI showed the most consistent inverse association across studies, whereas findings for uPDI were more heterogeneous (Table 2; Figs. 6, 7 and 8, Supplementary Figs. 5 to 7).

Results from case-control studies were generally consistent with findings from cohort studies, although effect estimates were larger and more variable [52, 56–58] (Supplementary Table 2). Most of these studies reported strong inverse associations between hPDI and colorectal cancer risk and positive associations with uPDI, particularly in hospital-based settings. Studies from China and Italy reported lower odds of colorectal cancer for the highest versus lowest hPDI categories, alongside markedly higher risks associated with uPDI [56, 57]. Two case–control studies additionally reported separate estimates for colon and rectal cancer, with consistent inverse associations for hPDI and positive associations for uPDI across both subsites [56, 57] (Supplementary Table 2).

Taken together, evidence from prospective cohort studies supports a modest protective association between higher adherence to overall and healthful plant-based dietary patterns and colorectal cancer risk, while evidence for unhealthy plant-based diets is less consistent. Findings from case–control studies point in a similar direction but should be interpreted with caution due to their higher susceptibility to recall and selection bias.

CI: confidence interval, HR: hazard ratio, SE: standard error. I² represents the percentage of total variation across studies attributable to between-study heterogeneity; values > 50% indicate substantial heterogeneity. τ² represents the estimate of between-study variance in the random-effects model.

CI: confidence interval, HR: hazard ratio, SE: standard error. I² represents the percentage of total variation across studies attributable to between-study heterogeneity; values > 50% indicate substantial heterogeneity. τ² represents the estimate of between-study variance in the random-effects model.

CI: confidence interval, HR: hazard ratio, SE: standard error. I² represents the percentage of total variation across studies attributable to between-study heterogeneity; values > 50% indicate substantial heterogeneity. τ² represents the estimate of between-study variance in the random-effects model.

### Prostate cancer

Three cohort studies examined the association between plant-based dietary indices and prostate cancer risk [29, 31, 37, 47]. Overall, no consistent associations were observed between adherence to plant-based dietary indices and prostate cancer incidence across individual cohort studies.

**Fig. 9:**
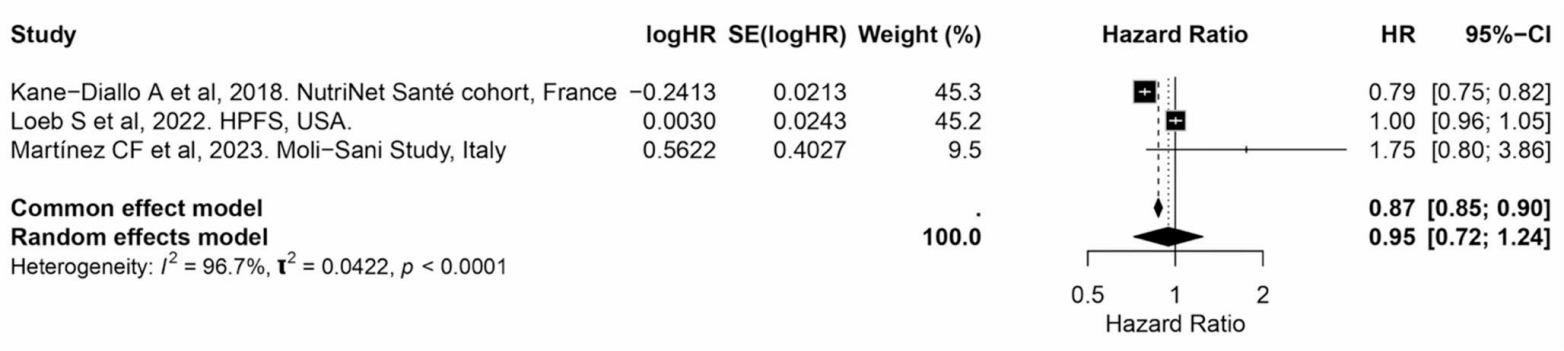
Meta-analysis of cohort studies assessing plant-based/pro-vegetarian dietary index (PDI/PVG) and prostate cancer risk: forest plot showing pooled hazard ratios for 10-unit increase in index

In the meta-analysis, no significant association was observed for overall or healthful plant-based diet adherence, either in categorical comparisons or per 10-unit increase [pooled HR per 10-unit increase in PDI: 0.95 (95% CI: 0.72, 1.24)] (Table 2; Fig. 9 and Supplementary Fig. 8 and 9). Considerable heterogeneity was present in the continuous analysis per 10-unit increase in PDI (I² = 96.0%), reflecting variability in study populations, exposure definitions, and outcome assessment.

Evidence from case-control studies was limited to a single hospital-based study conducted in Iran, which reported a substantially lower prostate cancer risk among participants with higher adherence to a PVG [50].

CI: confidence interval, HR: hazard ratio, SE: standard error. I² represents the percentage of total variation across studies attributable to between-study heterogeneity; values > 50% indicate substantial heterogeneity. τ² represents the estimate of between-study variance in the random-effects model. PDI and PVG were considered equivalent exposures due to their comparable construct and scoring.

### Lung cancer

Three cohort studies examined the association between plant-based dietary indices and lung cancer risk [29, 40, 46], reporting consistent inverse associations with overall plant-based diet (PVG/PDI). Only one reported estimates for healthy and unhealthy PDI, showing null associations with hPDI but significant higher risk associated with adherence to uPDI [46] (Supplementary Table 5).

**Fig. 10:**
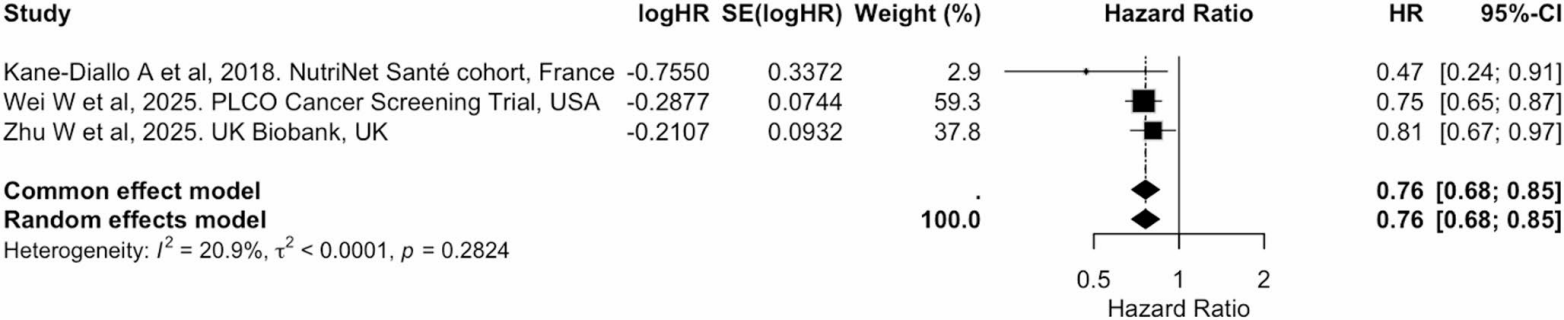
Meta-analysis of cohort studies assessing plant-based/pro-vegetarian dietary index (PDI/PVG) and lung cancer risk: forest plot showing pooled hazard ratios for the highest versus lowest index category

Due to lack of data for standardization, it was not possible to perform a meta-analysis per 10-unit increase. However, the pooled analysis of highest vs. lowest category of PVG/PDI estimates showed a significant 26% lower risk (pooled HR: 0.76 (95% CI: 0.68, 0.85), with low heterogeneity detected (I² = 20.9%) (Table 2; Fig. 10).

CI: confidence interval, HR: hazard ratio, SE: standard error. I² represents the percentage of total variation across studies attributable to between-study heterogeneity; values > 50% indicate substantial heterogeneity. τ² represents the estimate of between-study variance in the random-effects model. PDI and PVG were considered equivalent exposures due to their comparable construct and scoring.

### Liver cancer

**Fig. 11:**
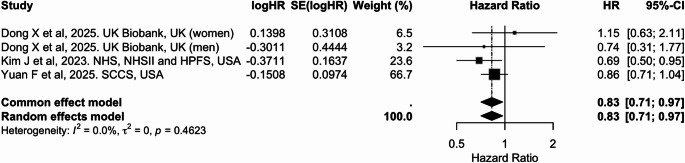
Meta-analysis of cohort studies assessing plant-based/pro-vegetarian dietary index (PDI/PVG) and liver cancer risk: forest plot showing pooled hazard ratios for 10-unit increase in index

**Fig. 12:**
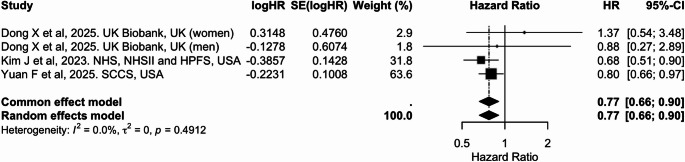
Meta-analysis of cohort studies assessing healthful plant-based dietary index (hPDI) and liver cancer risk: forest plot showing pooled hazard ratios for 10-unit increase in index

**Fig. 13:**
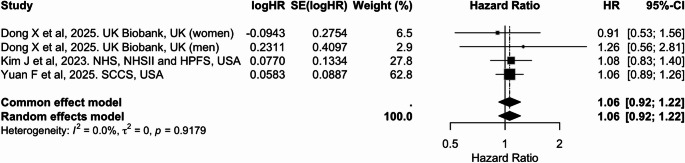
Meta-analysis of cohort studies assessing unhealthful plant-based dietary index (uPDI) and liver cancer risk: forest plot showing pooled hazard ratios for 10-unit increase in index

Four cohort studies contributed to the meta-analysis of liver cancer risk, including two large U.S. cohorts (NHS/NHSII/HPFS and SCCS) and sex-specific estimates from the UK Biobank, which were treated as independent contributions [30, 42, 44]. In the pooled analysis (Table 2 and Figs. 11, 12 and 13), higher adherence to an overall plant-based diet was associated with a significantly lower risk of liver cancer, with a pooled HR of 0.83 (95% CI: 0.71, 0.97) per 10-unit increase in PDI (Fig. 11). Similarly, hPDI showed a strong inverse association [0.77 (0.66, 0.90)], whereas no significant association was observed for uPDI (Figs. 12 and 13). No heterogeneity was detected across studies.

Two additional cohort studies evaluated hepatocellular carcinoma risk specifically but were not pooled with overall liver cancer outcomes due to differences in disease etiology and outcome definition [39, 43]. These studies consistently reported inverse associations for PDI and hPDI, and null or positive associations for uPDI (Supplementary Table 5).

CI: confidence interval, HR: hazard ratio, SE: standard error. I² represents the percentage of total variation across studies attributable to between-study heterogeneity; values > 50% indicate substantial heterogeneity. τ² represents the estimate of between-study variance in the random-effects model. PDI and PVG were considered equivalent exposures due to their comparable construct and scoring.

CI: confidence interval, HR: hazard ratio, SE: standard error. I² represents the percentage of total variation across studies attributable to between-study heterogeneity; values > 50% indicate substantial heterogeneity. τ² represents the estimate of between-study variance in the random-effects model.

CI: confidence interval, HR: hazard ratio, SE: standard error. I² represents the percentage of total variation across studies attributable to between-study heterogeneity; values > 50% indicate substantial heterogeneity. τ² represents the estimate of between-study variance in the random-effects model.

### Pancreas cancer

Three cohort studies reported data on plant-based dietary indices and risk of pancreas cancer [30, 38, 45]. Two studies including participants from the USA (NHS, NHSII and HPFS, and PLCO cancer screening trial) were consistent in the inverse associations reported for overall PDI, despite one of them reporting per 10-unit increase [30], and the other one per highest vs. lowest category [38] (Supplementary Table 5). Due to differences in estimate reporting and lack of data for standardization, it was not possible to perform a meta-analysis.

Evidence from two case-control studies showed inverse associations for healthy plant-based dietary indices and pancreas cancer risk [15, 56]. However, results were inconsistent for overall and unhealthy indices (Supplementary Table 1).

### Upper gastrointestinal cancers (oral cavity/pharynx, esophagus, and stomach)

Several cohort and case–control studies assessed the association between plant-based dietary indices and upper gastrointestinal cancers, including cancers of the oral cavity and pharynx, esophagus, and stomach (Supplementary Tables 2 and 5).

Evidence from cohort studies was limited. A study including data from three large US cohorts reported inverse associations between PDI and oral cavity/pharyngeal cancer risk, with similar but weaker patterns observed for hPDI and no clear association for uPDI [30]. No significant associations were observed for esophageal cancer in cohort analyses (Supplementary Table 5).

In contrast, case–control studies conducted in Spain and Italy consistently reported inverse associations for healthy plant-based dietary patterns and increased risks associated with unhealthy plant-based diets across these cancer sites [15, 56]. For oral cavity/pharynx, esophageal and stomach cancers, higher adherence to hPDI or hPVG scores was associated with substantially lower risk, whereas uPDI was associated with increased risk, particularly in categorical comparisons. Overall, findings from case–control studies suggest a protective role of healthy plant-based diets for upper gastrointestinal cancers, although prospective evidence remains limited (**Supplementary Table 2**).

### Digestive and related cancers (excluding colorectal)

A small number of cohort studies examined other digestive cancers, including small intestine, biliary tract, accessory organ cancers, and aggregated digestive organ outcomes [29–31]. In these studies, higher adherence to overall or healthy plant-based diets was generally associated with lower cancer risk, whereas unhealthy plant-based diets showed null or positive associations (Supplementary Table 5).

### Other cancer sites

Evidence for other cancer types, including respiratory tract, genitourinary organs, brain and nervous system tumors, lymphatic and hematopoietic malignancies, glioma, and basal cell carcinoma, was sparse and derived primarily from individual cohort or hospital-based case–control studies [31, 51] (Supplementary Tables 2 and 5). Overall, results were inconsistent across studies and cancer sites. One case–control study reported strong inverse associations for PDI and hPDI and markedly increased risks associated with uPDI, for glioma [51]. However, these findings were not supported by prospective cohort data. Only one cohort study reported data on respiratory tract cancer risk, showing an inverse association per 1-SD increase in PDI and a positive association per 1-SD increase in uPDI [31].

### Sensitivity analyses

Leave-one-out sensitivity analyses indicated that the main findings were generally robust (Supplementary Table 6). For breast cancer, the inverse associations observed for PDI and hPDI were moderately influenced by the exclusion of the largest contributing cohort [33], with statistical significance attenuated in some scenarios, whereas results for uPDI showed greater variability. For colorectal cancer, pooled estimates for PDI and uPDI were sensitive to the removal of a specific dataset (women) [35]; in contrast, the inverse association between hPDI and colorectal cancer remained statistically significant across all leave-one-out analyses. No impact of individual studies was observed for prostate cancer. For liver cancer, the direction of associations was largely consistent, although statistical significance varied across leave-one-out analyses for PDI and hPDI. For lung cancer, the inverse association observed for PDI was attenuated when the largest contributing study was excluded [40], while the direction of the association remained consistent.

## Discussion

This systematic review and meta-analysis examined the association between plant-based diet patterns – assessed using established indices such as the plant-based diet index (PDI), healthful plant-based diet index (hPDI), and unhealthful plant-based diet index (uPDI) – and cancer-specific risk. By integrating evidence from nineteen prospective cohort studies and thirteen case-control studies, this work provides an updated and comprehensive synthesis of the literature, with a particular focus on site-specific cancer outcomes and on distinguishing between the quality of plant-based foods consumed. Meta-analyses were limited to prospective cohorts to reduce bias and strengthen causal inference.

Across cohort studies, the findings suggest that higher adherence to overall and healthful plant-based diets was generally associated with a lower risk of selected cancer sites, most consistently for breast, colorectal, and liver cancer. In contrast, greater adherence to unhealthful plant-based diets was associated with higher risk of breast and colorectal cancer. Findings from case–control studies were broadly directionally consistent with cohort evidence for some cancer types but showed greater variability and were therefore interpreted separately. These associations were more consistently observed in large prospective cohort studies conducted in the United States (e.g., NHS, NHS II, HPFS, PLCO, Multiethnic Cohort) and Europe (e.g., UK Biobank, SUN, E3N, NutriNet-Santé, Moli-Sani, EPIC), which enhances confidence in the internal validity of the findings across diverse populations. Nevertheless, variability across cancer sites, geographic regions, and dietary assessment methods indicates that results should be interpreted with caution. Overall, the recurrent pattern of inverse associations for healthful plant-based dietary patterns and positive associations for unhealthful plant-based dietary patterns across multiple cohorts supports a differentiated role of plant-based diet quality in cancer risk.

The growing popularity of dietary patterns that prioritize plant-derived foods – driven by ethical, environmental, and health-related considerations – has coincided with an increase in research interest over the past decade [60, 61]. Importantly, this body of research encompasses a broad range of dietary approaches, from vegetarian and vegan diets to plant-based patterns that emphasize plant-derived foods without excluding animal products. While earlier studies often treated plant-based diets as a homogeneous exposure, more recent approaches differentiate between healthful and unhealthful plant-based patterns using validated, structured indices [3, 6, 7, 62]. In this context, the present review extends prior literature by synthesizing cancer-specific evidence while explicitly accounting for the quality of plant-derived foods consumed.

Healthful plant-based diets emphasize minimally processed foods such as whole grains, fruits, vegetables, legumes, and nuts, which are rich in dietary fiber, micronutrients, and bioactive compounds, including (poly)phenols [63–65]. The inverse associations observed for hPDI plausibly reflect multiple biological pathways, including reduced systemic inflammation, improved insulin sensitivity, modulation of estrogen metabolism, and decreased oxidative stress [63, 66, 67]. For instance, dietary fiber enhances gut microbiota diversity and promotes the production of short-chain fatty acids, which play a key role in epithelial integrity and immune regulation – mechanisms particularly relevant for colorectal carcinogenesis [68, 69]. Additionally, (poly)phenols may play an anti-cancer role through pathways involving cell cycle regulation, angiogenesis inhibition, and apoptosis promotion [69]. Conversely, unhealthful plant-based diets are typically rich in refined grains, added sugars, and ultra-processed foods, and low in fiber and essential micronutrients [3, 7]. These dietary components have been linked to increased glycemic load, chronic inflammation, metabolic dysfunction and chronic diseases, which may increase susceptibility to carcinogenesis [70, 71]. Dysbiosis and immune impairment may further contribute to carcinogenesis, especially in obesity-related cancers [72]. Diet-induced adiposity and metabolic dysregulation may further exacerbate cancer risk through pathways involving hormonal dysregulation (e.g., elevated insulin-like growth factor 1 and estrogens), systemic inflammation, and insulin resistance, which are recognized mediators of cancer development [66, 72].

Overall, these findings are broadly consistent with prior reviews and meta-analyses linking high-quality plant-based diets and specific dietary components (e.g., fiber, whole grains, legumes) with lower cancer risk [12, 66, 73]. Importantly, the present work adds to this evidence base by highlighting the relevance of plant-based diet quality – rather than plant-food consumption per se – in shaping cancer risk.

Using the GRADE framework [23], the certainty of evidence ranged from moderate to very low across cancer sites and dietary indices. Moderate-certainty evidence supported an inverse association between healthful plant-based diets and breast and liver cancer risk, as well as overall plant-based diets and liver and lung cancer risk. Although future studies may influence the magnitude of these associations, the overall direction of effect is unlikely to change substantially. Evidence for other cancer sites remained limited by heterogeneity, imprecision, and residual bias, emphasizing the need for further high-quality prospective research.

From a public health nutrition perspective, these findings support dietary recommendations that promote predominantly plant-based eating patterns while emphasizing quality. The consistent inverse associations observed for healthful plant-based diets across several cancer sites suggest that cancer prevention strategies should go beyond encouraging higher plant food consumption per se and instead prioritize minimally processed plant foods. Conversely, the observed adverse associations with unhealthful plant-based dietary patterns highlight the need to limit refined grains, added sugars, and ultra-processed plant-based products. These results align with current cancer prevention guidelines [74] and underscore the importance of distinguishing between healthful and unhealthful plant-based patterns for cancer prevention strategies at the population level.

### Strengths and limitations

Several limitations should be considered when interpreting the findings of this review. All included studies were observational, which limits causal inference and leaves room for residual confounding, despite multivariable adjustments. The ROBINS-E assessment indicated an overall moderate risk of bias across cohort studies, primarily driven by residual confounding and limitations in dietary exposure assessment, which should be considered when interpreting the magnitude of the observed associations. Case-control studies provided complementary evidence; however, their findings should be interpreted cautiously given their greater susceptibility to reverse causation as well as recall and selection bias. Another limitation is the use of subjective dietary assessment tools, as included studies relied on food frequency questionnaires or 24-hour recalls, which are subject to measurement error and recall bias [75]. Even though most studies used validated instruments, misclassification of dietary exposure cannot be excluded. In addition, although some cohorts incorporated repeated dietary assessments and time-updated exposures, others relied on a single baseline dietary measurement. As a meta-analysis of published estimates, we were unable to harmonize exposure modeling across studies, which may contribute to residual heterogeneity. Most included cohort studies were conducted in Western countries and predominantly involved populations of European ancestry. Although some diversity was captured through studies such as the Multiethnic Cohort and the Singapore Chinese Health Study, evidence from low- and middle-income countries and non-Western dietary contexts remains limited. Plant-based dietary patterns may differ substantially across cultural, socioeconomic, and food-system environments, and associations observed in Western populations may not fully extrapolate to other global settings. Additional large-scale prospective studies in underrepresented populations are therefore needed to evaluate the consistency of these associations across diverse dietary and epidemiologic contexts. An additional limitation relates to the intrinsic assumptions of the plant-based dietary indices used. Although they have been widely applied and validated, the indices rely on predefined food group classifications that may not fully capture the heterogeneity of individual foods [3, 7]. For example, starchy vegetables were categorized as less healthful plant foods, which may result in lower scores for dietary patterns that include higher intakes of foods such as potatoes. While this classification reflects their higher glycemic load and frequent consumption in processed forms in Western diets, it may not fully account for differences in preparation methods or their contribution within balanced dietary patterns. As a result, some dietary components may be over- or under-weighted within the indices, potentially contributing to exposure misclassification. Another methodological limitation relates to the frequent use of categorical exposure modelling in the primary studies, which may lead to loss of information, reduced statistical power, and results that depend on arbitrary cut-points rather than underlying dose-response relationships [76]. In the present meta-analysis, this issue was partially addressed by standardizing estimates to a continuous 10-unit increment and, when possible, by applying dose–response modelling using generalized least squares regression. Nevertheless, residual bias related to exposure categorization cannot be fully excluded. Moderate to substantial heterogeneity was observed in some meta-analyses, likely due to differences in population characteristics, dietary assessment methods, index construction and scoring, and covariate adjustment strategies. In addition, the limited number of eligible cohort studies for certain cancer sites for meta-analysis restricted the possibility of conducting stratified analyses, as well as a formal assessment of publication bias. Funnel plot asymmetry and Egger’s regression test have limited statistical power when fewer than ten studies are available, and any apparent asymmetry may reflect random variation rather than true reporting bias [24].

This review also has several strengths. The standardization of effect estimates to a 10-unit increase in dietary indices enhanced comparability across studies and facilitated dose-response interpretations [22]. The inclusion of large prospective cohorts with long follow-up periods enhances internal validity and reduces the likelihood of reverse causation. The use of validated plant-based dietary indices provides a more accurate reflection of overall diet quality than individual food or nutrient-based approaches, allowing for a more comprehensive evaluation of healthful and unhealthful plant-based diets in relation to cancer risk.

## Conclusion

This systematic review and meta-analysis provides comprehensive evidence that the quality of plant-based dietary patterns is an important determinant of cancer risk. Higher adherence to overall and healthful plant-based diet indices was associated with a lower risk of several site-specific cancers, including breast, colorectal, liver, lung, and pancreatic cancer, whereas greater adherence to unhealthful plant-based diets was associated with an increased cancer risk for multiple sites. These findings highlight that not all plant-based diets confer similar health benefits and underscore the relevance of distinguishing between healthful and unhealthful plant foods when evaluating cancer risk.

The associations were most robust in large prospective cohort studies and were supported by moderate certainty of evidence, although residual confounding and heterogeneity cannot be fully excluded due to the observational nature of the evidence. Case-control studies provided complementary support but were not quantitatively synthesized because of their greater susceptibility to bias.

From a public health perspective, these findings support dietary recommendations that emphasize the quality of plant-based foods rather than the exclusion of animal products per se. As plant-based diets continue to gain popularity, encouraging incremental shifts toward diets rich in high-quality, minimally processed plant foods while limiting refined and ultra-processed plant foods may represent a practical and effective strategy for cancer risk reduction. Future large-scale prospective studies in more diverse populations and non-Western dietary contexts are needed to further clarify these associations and strengthen the evidence base for tailored cancer prevention strategies.

## Supplementary Information

Below is the link to the electronic supplementary material.

Supplementary Material 1

## Data Availability

The data used in this review come from published articles, all of which are identified in the References section.
