# Supplementary material for: Healthful and unhealthful plant-based diets and site-specific cancer risk: a systematic review and meta-analysis of observational studies
Source: Eur J Nutr. 2026 Apr 21;65(4):121. doi: 10.1007/s00394-026-03948-2 (PMC13099660; doi:10.1007/s00394-026-03948-2)
Supplement: Supplementary file 1 — Supplementary Material 1 [file 394_2026_3948_MOESM1_ESM.pdf]

Healthful and Unhealthful Plant-Based Diets and Site-specific Cancer Risk: A Systematic Review and Meta-Analysis of Observational Studies

European Journal of Nutrition

Mercedes Gil-Lespinard, Lucía Iglesias-Vázquez, Paula Jakszyn
Corresponding author: Dr Paula Jakszyn paujak@iconcologia.net

Supplementary materials

Content

Supplementary Table 1. PRISMA 2020 Checklist 2

Supplementary Table 2. Results from included case-control studies. 6

Supplementary Table 3. Quality assessment according to the Newcastle-Ottawa Scale for case-control studies. 11

Supplementary Table 4. Certainty of the evidence using the GRADE approach. 12

Supplementary Table 5. Association between plant-based dietary indices and cancer risk in prospective cohort studies 13

Supplementary Table 6. Results of leave-one-out sensitivity analyses assessing the robustness of meta-analytic estimates 21

Supplementary Figure 1. Quality of included cohort studies (n=19) using the ROBINS-E tool. 24

Supplementary Figure 2. Meta-analysis of cohort studies assessing plant-based/pro-vegetarian dietary index (PDI/PVG) and breast cancer risk: forest plot showing pooled hazard ratios for the highest versus lowest index category. 26

Supplementary Figure 3. Meta-analysis of cohort studies assessing healthful plant-based dietary index (hPDI) and breast cancer risk: forest plot showing pooled hazard ratios for the highest versus lowest index category 27

Supplementary Figure 4. Meta-analysis of cohort studies assessing unhealthful plant-based dietary index (uPDI) and breast cancer risk: forest plot showing pooled hazard ratios for the highest versus lowest index category. 28

Supplementary Figure 5. Meta-analysis of cohort studies assessing plant-based/pro-vegetarian dietary index (PDI/PVG) and colorectal cancer risk: forest plot showing pooled hazard ratios for the highest versus lowest index category. 29

Supplementary Figure 6. Meta-analysis of cohort studies assessing healthful plant-based dietary index (hPDI) and colorectal cancer risk: forest plot showing pooled hazard ratios for the highest versus lowest index category. 30

Supplementary Figure 7. Meta-analysis of cohort studies assessing unhealthful plant-based dietary index (uPDI) and colorectal cancer risk: forest plot showing pooled hazard ratios for the highest versus lowest index category. 31

Supplementary Figure 8. Meta-analysis of cohort studies assessing plant-based/pro-vegetarian dietary index (PDI/PVG) and prostate cancer risk: forest plot showing pooled hazard ratios for the highest versus lowest index category. 32

Supplementary Figure 9. Meta-analysis of cohort studies assessing plant-based/pro-vegetarian dietary index (PDI/PVG) and prostate cancer risk: forest plot showing pooled hazard ratios for the highest versus lowest index category. 33

Supplementary Table 1. PRISMA 2020 Checklist

Section and Topic	Item #	Checklist item	Location where item is reported (page#)
**TITLE**			
Title	1	Identify the report as a systematic review.	Page 1
**ABSTRACT**			
Abstract	2	See the PRISMA 2020 for Abstracts checklist.	Page 2, lines 1-22
**INTRODUCTION**			
Rationale	3	Describe the rationale for the review in the context of existing knowledge.	Page 4, lines 25-65
Objectives	4	Provide an explicit statement of the objective(s) or question(s) the review addresses.	Page 5, lines 59-65
**METHODS**			
Eligibility criteria	5	Specify the inclusion and exclusion criteria for the review and how studies were grouped for the syntheses.	Pages 5-7, lines 67-109, Figure 1
Information sources	6	Specify all databases, registers, websites, organisations, reference lists and other sources searched or consulted to identify studies. Specify the date when each source was last searched or consulted.	Page 6, lines 78-92
Search strategy	7	Present the full search strategies for all databases, registers and websites, including any filters and limits used.	Pages 6-7, lines 78-102
Selection process	8	Specify the methods used to decide whether a study met the inclusion criteria of the review, including how many reviewers screened each record and each report retrieved, whether they worked independently, and if applicable, details of automation tools used in the process.	Pages 6-7, lines 78-102
Data collection process	9	Specify the methods used to collect data from reports, including how many reviewers collected data from each report, whether they worked independently, any processes for obtaining or confirming data from study investigators, and if applicable, details of automation tools used in the process.	Pages 6-7, lines 88-102
Data items	10a	List and define all outcomes for which data were sought. Specify whether all results that were compatible with each outcome domain in each study were sought (e.g. for all measures, time points, analyses), and if not, the methods used to decide which results to collect.	NA
	10b	List and define all other variables for which data were sought (e.g. participant and intervention characteristics, funding sources). Describe any assumptions made about any missing or unclear information.	NA
Study risk of bias assessment	11	Specify the methods used to assess risk of bias in the included studies, including details of the tool(s) used, how many reviewers assessed each study and whether they worked independently, and if applicable, details of automation tools used in the process.	Pages 7-8, lines 110-

Section and Topic	Item #	Checklist item	Location where item is reported (page#)
			119
Effect measures	12	Specify for each outcome the effect measure(s) (e.g. risk ratio, mean difference) used in the synthesis or presentation of results.	Page 7, lines 108-109
Synthesis methods	13a	Describe the processes used to decide which studies were eligible for each synthesis (e.g. tabulating the study intervention characteristics and comparing against the planned groups for each synthesis (item #5)).	NA
	13b	Describe any methods required to prepare the data for presentation or synthesis, such as handling of missing summary statistics, or data conversions.	Page 8, lines 127-138
	13c	Describe any methods used to tabulate or visually display results of individual studies and syntheses.	NA
	13d	Describe any methods used to synthesize results and provide a rationale for the choice(s). If meta-analysis was performed, describe the model(s), method(s) to identify the presence and extent of statistical heterogeneity, and software package(s) used.	Page 8-9, lines 120-147
	13e	Describe any methods used to explore possible causes of heterogeneity among study results (e.g. subgroup analysis, meta-regression).	NA
	13f	Describe any sensitivity analyses conducted to assess robustness of the synthesized results.	Page 9, lines 144-147
Reporting bias assessment	14	Describe any methods used to assess risk of bias due to missing results in a synthesis (arising from reporting biases).	NA
Certainty assessment	15	Describe any methods used to assess certainty (or confidence) in the body of evidence for an outcome.	NA
**RESULTS**			
Study selection	16a	Describe the results of the search and selection process, from the number of records identified in the search to the number of studies included in the review, ideally using a flow diagram.	Page 9, lines 150-158 and Figure 2
	16b	Cite studies that might appear to meet the inclusion criteria, but which were excluded, and explain why they were excluded.	NA
Study characteristics	17	Cite each included study and present its characteristics.	Pages 10-11, lines 160-189, Tables 1, S2 and S5
Risk of bias in	18	Present assessments of risk of bias for each included study.	Pages 13-

Section and Topic	Item #	Checklist item	Location where item is reported (page#)
studies			14, lines 191-211, Figure S1 Table S3
Results of individual studies	19	For all outcomes, present, for each study: (a) summary statistics for each group (where appropriate) and (b) an effect estimate and its precision (e.g. confidence/credible interval), ideally using structured tables or plots.	Tables 1, S2, S5
Results of syntheses	20a	For each synthesis, briefly summarise the characteristics and risk of bias among contributing studies.	Pages 13-14, lines 191-211
	20b	Present results of all statistical syntheses conducted. If meta-analysis was done, present for each the summary estimate and its precision (e.g. confidence/credible interval) and measures of statistical heterogeneity. If comparing groups, describe the direction of the effect.	Pages 14-25, lines 212-433, Table 2, Figures 3-13, Figures S2-S8
	20c	Present results of all investigations of possible causes of heterogeneity among study results.	NA
	20d	Present results of all sensitivity analyses conducted to assess the robustness of the synthesized results.	Page 25, lines 423-433. Table S6
Reporting biases	21	Present assessments of risk of bias due to missing results (arising from reporting biases) for each synthesis assessed.	NA
Certainty of evidence	22	Present assessments of certainty (or confidence) in the body of evidence for each outcome assessed.	NA
**DISCUSSION**			
Discussion	23a	Provide a general interpretation of the results in the context of other evidence.	Pages 25-28, lines 435-504
	23b	Discuss any limitations of the evidence included in the review.	Pages 28-30, lines 505-556
	23c	Discuss any limitations of the review processes used.	Pages 28-30, lines 505-556

Section and Topic	Item #	Checklist item	Location where item is reported (page#)
	23d	Discuss implications of the results for practice, policy, and future research.	Page 28, lines 496-504
**OTHER INFORMATION**			
Registration and protocol	24a	Provide registration information for the review, including register name and registration number, or state that the review was not registered.	Pages 5-6, lines 83-85
	24b	Indicate where the review protocol can be accessed, or state that a protocol was not prepared.	Pages 5-6, lines 83-85
	24c	Describe and explain any amendments to information provided at registration or in the protocol.	NA
Support	25	Describe sources of financial or non-financial support for the review, and the role of the funders or sponsors in the review.	Page 31, line 580
Competing interests	26	Declare any competing interests of review authors.	Page 31, line 579
Availability of data, code and other materials	27	Report which of the following are publicly available and where they can be found: template data collection forms; data extracted from included studies; data used for all analyses; analytic code; any other materials used in the review.	Page 32, lines 586-587

From: Page MJ, McKenzie JE, Bossuyt PM, Boutron I, Hoffmann TC, Mulrow CD, et al. The PRISMA 2020 statement: an updated guideline for reporting systematic reviews. BMJ 2021;372:n71. doi: 10.1136/bmj.n71. This work is licensed under CC BY 4.0. To view a copy of this license, visit https://creativecommons.org/licenses/by/4.0/

Supplementary Table 2. Results from included case-control studies.

Author, year	Study base, country	Cancer site	Dietary index	Exposure contrast	OR (95% CI)
Hosseini Y et al, 2023	Hospital-based, Iran	Breast	PVG	Highest vs lowest category	0.49 (0.27, 0.88)
Payandeh N et al, 2021	Hospital-based, Iran	Breast	PDI	Highest vs lowest category	1.00 (0.55, 1.83)
Payandeh N et al, 2021	Hospital-based, Iran	Breast	hPDI	Highest vs lowest category	0.89 (0.49, 1.62)
Payandeh N et al, 2021	Hospital-based, Iran	Breast	uPDI	Highest vs lowest category	1.80 (0.95, 3.42)
Rigi S et al, 2021	Hospital-based, Iran	Breast	PDI	Highest vs lowest category	0.33 (0.22, 0.50)
Rigi S et al, 2021	Hospital-based, Iran	Breast	hPDI	Highest vs lowest category	0.64 (0.43, 0.94)
Rigi S et al, 2021	Hospital-based, Iran	Breast	uPDI	Highest vs lowest category	2.23 (1.48, 3.36)
Sasanfar B et al, 2021	Hospital-based, Iran	Breast	PDI	Highest vs lowest category	0.96 (0.65, 1.42)
Sasanfar B et al, 2022	Hospital-based, Iran	Breast	hPDI	Highest vs lowest category	0.61 (0.40, 0.93)
Sasanfar B et al, 2023	Hospital-based, Iran	Breast	uPDI	Highest vs lowest category	1.21 (0.80, 1.84)
Souni F et al, 2025	Hospital-based, Iran	Breast	PDI	Highest vs lowest category	1.05 (0.58, 1.90)
Souni F et al, 2025	Hospital-based, Iran	Breast	hPDI	Highest vs lowest category	0.49 (0.27, 0.89)
Souni F et al, 2025	Hospital-based, Iran	Breast	uPDI	Highest vs lowest category	1.43 (0.79, 2.58)
Nejad E et al, 2023	Hospital-based, Iran	Colorectum	PVG	Highest vs lowest category	0.35 (0.14, 0.87)
Turati F et al, 2025	Hospital-based, Italy	Colorectum	PDI	Highest vs lowest category	0.87 (0.71, 1.06)
Turati F et al, 2025	Hospital-based, Italy	Colorectum	PDI	Per 3-unit increase	0.97 (0.94, 1.01)
Turati F et al, 2025	Hospital-based, Italy	Colorectum	hPDI	Highest vs lowest category	0.69 (0.57, 0.84)
Turati F et al, 2025	Hospital-based, Italy	Colorectum	hPDI	Per 3-unit increase	0.93 (0.89, 0.96)
Turati F et al, 2025	Hospital-based, Italy	Colorectum	uPDI	Highest vs lowest category	2.28 (1.86, 2.81)
Turati F et al, 2025	Hospital-based, Italy	Colorectum	uPDI	Per 3-unit increase	1.13 (1.10, 1.17)
Wu B et al, 2022	Population-based, China	Colorectum	PDI	Highest vs lowest category	0.79 (0.66, 0.95)
Wu B et al, 2022	Population-based, China	Colorectum	hPDI	Highest vs lowest category	0.45 (0.38, 0.55)

Author, year	Study base, country	Cancer site	Dietary index	Exposure contrast	OR (95% CI)
Wu B et al, 2022	Population-based, China	Colorectum	uPDI	Highest vs lowest category	1.45 (1.18, 1.78)
Yarmand S et al, 2024	Hospital-based, Iran	Colorectum	PDI	Highest vs lowest category	0.92 (0.37, 2.29)
Yarmand S et al, 2024	Hospital-based, Iran	Colorectum	hPDI	Highest vs lowest category	0.21 (0.07, 0.56)
Yarmand S et al, 2024	Hospital-based, Iran	Colorectum	uPDI	Highest vs lowest category	6.76 (2.41, 18.9)
Turati F et al, 2025	Hospital-based, Italy	Colon	PDI	Highest vs lowest category	0.87 (0.71, 1.06)
Turati F et al, 2025	Hospital-based, Italy	Colon	PDI	Per 3-unit increase	0.98 (0.94, 1.02)
Turati F et al, 2025	Hospital-based, Italy	Colon	hPDI	Highest vs lowest category	0.69 (0.57, 0.84)
Turati F et al, 2025	Hospital-based, Italy	Colon	hPDI	Per 3-unit increase	0.93 (0.89, 0.96)
Turati F et al, 2025	Hospital-based, Italy	Colon	uPDI	Highest vs lowest category	2.28 (1.86, 2.81)
Turati F et al, 2025	Hospital-based, Italy	Colon	uPDI	Per 3-unit increase	1.15 (1.11, 1.20)
Wu B et al, 2022	Population-based, China	Colon	PDI	Highest vs lowest category	0.81 (0.66, 1.00)
Wu B et al, 2022	Population-based, China	Colon	hPDI	Highest vs lowest category	0.43 (0.34, 0.53)
Wu B et al, 2022	Population-based, China	Colon	uPDI	Highest vs lowest category	1.45 (1.15, 1.82)
Turati F et al, 2025	Hospital-based, Italy	Rectum	PDI	Highest vs lowest category	0.80 (0.60, 1.06)
Turati F et al, 2025	Hospital-based, Italy	Rectum	PDI	Per 3-unit increase	0.95 (0.90, 1.00)
Turati F et al, 2025	Hospital-based, Italy	Rectum	hPDI	Highest vs lowest category	0.71 (0.54, 0.94)
Turati F et al, 2025	Hospital-based, Italy	Rectum	hPDI	Per 3-unit increase	0.93 (0.89, 0.98)
Turati F et al, 2025	Hospital-based, Italy	Rectum	uPDI	Highest vs lowest category	1.94 (1.44, 2.60)
Turati F et al, 2025	Hospital-based, Italy	Rectum	uPDI	Per 3-unit increase	1.10 (1.05, 1.15)
Wu B et al, 2022	Population-based, China	Rectum	PDI	Highest vs lowest category	0.80 (0.62, 1.02)
Wu B et al, 2022	Population-based, China	Rectum	hPDI	Highest vs lowest category	0.50 (0.38, 0.64)
Wu B et al, 2022	Population-based, China	Rectum	uPDI	Highest vs lowest category	1.41 (1.06, 1.88)
Oncina-Cánovas A et al, 2022	Hospital-based, Spain	Esophagus	PVG	Highest vs lowest category	0.37 (0.32, 0.42)

Author, year	Study base, country	Cancer site	Dietary index	Exposure contrast	OR (95% CI)
Oncina-Cánovas A et al, 2022	Hospital-based, Spain	Esophagus	PVG	Per 1-unit increase	0.94 (0.91, 0.97)
Oncina-Cánovas A et al, 2022	Hospital-based, Spain	Esophagus	hPVG	Highest vs lowest category	0.72 (0.58, 0.90)
Oncina-Cánovas A et al, 2022	Hospital-based, Spain	Esophagus	hPVG	Per 1-unit increase	0.98 (0.95, 1.00)
Oncina-Cánovas A et al, 2022	Hospital-based, Spain	Esophagus	uPVG	Highest vs lowest category	1.26 (1.00, 1.60)
Oncina-Cánovas A et al, 2022	Hospital-based, Spain	Esophagus	uPVG	Per 1-unit increase	1.01 (0.99, 1.03)
Turati F et al, 2025	Hospital-based, Italy	Esophagus	PDI	Highest vs lowest category	0.47 (0.31, 0.72)
Turati F et al, 2025	Hospital-based, Italy	Esophagus	PDI	Per 3-unit increase	0.81 (0.74, 0.89)
Turati F et al, 2025	Hospital-based, Italy	Esophagus	hPDI	Highest vs lowest category	0.59 (0.39, 0.91)
Turati F et al, 2025	Hospital-based, Italy	Esophagus	hPDI	Per 3-unit increase	0.90 (0.82, 0.98)
Turati F et al, 2025	Hospital-based, Italy	Esophagus	uPDI	Highest vs lowest category	1.34 (0.87, 2.05)
Turati F et al, 2025	Hospital-based, Italy	Esophagus	uPDI	Per 3-unit increase	1.07 (0.98, 1.17)
Oncina-Cánovas A et al, 2022	Hospital-based, Spain	Stomach	PVG	Highest vs lowest category	0.34 (0.27, 0.43)
Oncina-Cánovas A et al, 2022	Hospital-based, Spain	Stomach	PVG	Per 1-unit increase	0.94 (0.92, 0.96)
Oncina-Cánovas A et al, 2022	Hospital-based, Spain	Stomach	hPVG	Highest vs lowest category	0.72 (0.58, 0.90)
Oncina-Cánovas A et al, 2022	Hospital-based, Spain	Stomach	hPVG	Per 1-unit increase	0.98 (0.95, 1.00)
Oncina-Cánovas A et al, 2022	Hospital-based, Spain	Stomach	uPVG	Highest vs lowest category	1.26 (1.00, 1.60)
Oncina-Cánovas A et al, 2022	Hospital-based, Spain	Stomach	uPVG	Per 1-unit increase	1.01 (0.99, 1.03)
Turati F et al, 2025	Hospital-based, Italy	Stomach	PDI	Highest vs lowest category	0.70 (0.46, 1.09)
Turati F et al, 2025	Hospital-based, Italy	Stomach	PDI	Per 3-unit increase	0.96 (0.87, 1.06)
Turati F et al, 2025	Hospital-based, Italy	Stomach	hPDI	Highest vs lowest category	0.42 (0.27, 0.67)
Turati F et al, 2025	Hospital-based, Italy	Stomach	hPDI	Per 3-unit increase	0.84 (0.77, 0.92)
Turati F et al, 2025	Hospital-based, Italy	Stomach	uPDI	Highest vs lowest category	1.46 (0.96, 2.24)
Turati F et al, 2025	Hospital-based, Italy	Stomach	uPDI	Per 3-unit increase	1.12 (1.03, 1.23)

Author, year	Study base, country	Cancer site	Dietary index	Exposure contrast	OR (95% CI)
Oncina-Cánovas A et al, 2022	Hospital-based, Spain	Pancreas	PVG	Highest vs lowest category	0.43 (0.35, 0.52)
Oncina-Cánovas A et al, 2022	Hospital-based, Spain	Pancreas	PVG	Per 1-unit increase	0.95 (0.92, 0.98)
Oncina-Cánovas A et al, 2022	Hospital-based, Spain	Pancreas	hPVG	Highest vs lowest category	0.74 (0.59, 0.92)
Oncina-Cánovas A et al, 2022	Hospital-based, Spain	Pancreas	hPVG	Per 1-unit increase	0.98 (0.96, 1.00)
Oncina-Cánovas A et al, 2022	Hospital-based, Spain	Pancreas	uPVG	Highest vs lowest category	0.91 (0.72, 1.14)
Oncina-Cánovas A et al, 2022	Hospital-based, Spain	Pancreas	uPVG	Per 1-unit increase	0.98 (0.96, 1.00)
Turati F et al, 2025	Hospital-based, Italy	Pancreas	PDI	Highest vs lowest category	0.77 (0.52, 1.15)
Turati F et al, 2025	Hospital-based, Italy	Pancreas	PDI	Per 3-unit increase	0.95 (0.87, 1.04)
Turati F et al, 2025	Hospital-based, Italy	Pancreas	hPDI	Highest vs lowest category	0.60 (0.41, 0.89)
Turati F et al, 2025	Hospital-based, Italy	Pancreas	hPDI	Per 3-unit increase	0.88 (0.81, 0.96)
Turati F et al, 2025	Hospital-based, Italy	Pancreas	uPDI	Highest vs lowest category	1.74 (1.14, 2.65)
Turati F et al, 2025	Hospital-based, Italy	Pancreas	uPDI	Per 3-unit increase	1.10 (1.02, 1.20)
Leone A et al, 2020	Population-based, Spain	Basal cell carcinoma	PVG	Highest vs lowest category	1.54 (0.61, 3.91)
Mahmoodi et al, 2024	Hospital-based, Iran	Prostate	PVG	Highest vs lowest category	0.34 (0.15, 0.75)
Mousavi S et al, 2021	Hospital-based, Iran	Glioma	PDI	Highest vs lowest category	0.45 (0.25, 0.82)
Mousavi S et al, 2022	Hospital-based, Iran	Glioma	hPDI	Highest vs lowest category	0.28 (0.14, 0.55)
Mousavi S et al, 2023	Hospital-based, Iran	Glioma	uPDI	Highest vs lowest category	4.89 (2.33, 10.28)
Turati F et al, 2025	Hospital-based, Italy	Oral cavity/pharynx	PDI	Highest vs lowest category	0.63 (0.47, 0.84)
Turati F et al, 2025	Hospital-based, Italy	Oral cavity/pharynx	PDI	Per 3-unit increase	0.92 (0.87, 0.97)
Turati F et al, 2025	Hospital-based, Italy	Oral cavity/pharynx	hPDI	Highest vs lowest category	0.52 (0.39, 0.70)
Turati F et al, 2025	Hospital-based, Italy	Oral cavity/pharynx	hPDI	Per 3-unit increase	0.89 (0.85, 0.93)
Turati F et al, 2025	Hospital-based, Italy	Oral cavity/pharynx	uPDI	Highest vs lowest category	1.43 (1.06, 1.94)
Turati F et al, 2025	Hospital-based, Italy	Oral cavity/pharynx	uPDI	Per 3-unit increase	1.07 (1.01, 1.12)

Author, year	Study base, country	Cancer site	Dietary index	Exposure contrast	OR (95% CI)

OR: Odds Ratio, CI: confidence interval. Dietary indices: PDI: plant-based dietary pattern, hPDI: healthful PDI, uPDI: unhealthful PDI, PVG: pro-vegetarian dietary pattern, hPVG: healthful PVG; uPVG: unhealthful PVG. OR and 95% CI correspond to the most fully-adjusted model. Covariate adjustment was generally comprehensive, including age, sex, body mass index, smoking status, education, physical activity, and total energy intake.

Supplementary Table 3. Quality assessment according to the Newcastle-Ottawa Scale for case-control studies.

Study	Selection	Comparability	Exposure	Score (0-9)
Hosseini Y et al, 2023. Iran	+ + +	+ +	+ +	7
Leone A et al, 2020. Spain	+ + +	+ +	+ + +	8
Mahmoodi et al, 2024. Iran	+ + + +	+	+ +	7
Mousavi S et al, 2021. Iran	+ + +	+ +	+ +	7
Nejad E et al, 2023. Iran	+ + +	+ +	+ +	7
Oncina-Canovas et al, 2022. Spain	+ + + +	+ +	+ +	8
Payandeh N et al, 2021. Iran	+ + +	+ +	+ +	7
Rigi S et al, 2021. Iran	+ + + +	+ +	+ +	8
Sansafar B et al, 2021. Iran	+ + +	+ +	+ +	7
Souni F et al, 2025. Iran	+ + + +	+ +	+ + +	9
Turati F et al, 2025. Italy	+ + + +	+ +	+ +	8
Wu B et al, 2022. China	+ + +	+ +	+ +	7
Yarmand S et al, 2024. Iran	+ + +	+ +	+ +	7

Semi-quantitative assessment of study methodology quality using a star system, adapted from the Newcastle-Ottawa Scale. Each item included the following subcategories: Selection (0-4 points): Adequation of case definition, representativeness of the cases, selection of controls, definition of controls; Comparability (0-3 points): Comparability of cases and controls based on the design or analysis, ascertainment of exposure; Exposure (0-2 points): Same method of ascertainment for cases and controls, non-response rate.

Supplementary Table 4. Certainty of the evidence using the GRADE approach.

Cancer site	Dietary index	Studies (k)	Pooled HR (95% CI)	I^2 (%)	Risk of bias	Inconsistency	Indirectness	Imprecision	Publication bias	Certainty
Breast	PDI	5	0.92 (0.87, 0.98)	57.5	Serious	Serious	Not serious	Not serious	Undetected	Low
Breast	hPDI	5	0.94 (0.93, 0.96)	20.4	Serious	Not serious	Not serious	Not serious	Undetected	Moderate
Breast	uPDI	5	1.03 (1.01, 1.06)	48.6	Serious	Serious	Not serious	Not serious	Undetected	Low
Colorectum	PDI	5	0.95 (0.91, 0.98)	66.7	Serious	Serious	Not serious	Not serious	Undetected	Low
Colorectum	hPDI	5	0.95 (0.92, 0.98)	55.2	Serious	Serious	Not serious	Not serious	Undetected	Low
Colorectum	uPDI	5	1.03 (0.99, 1.06)	63.1	Serious	Serious	Not serious	Serious	Undetected	Very low
Prostate	PDI	3	0.95 (0.72, 1.24)	96.7	Serious	Serious	Not serious	Serious	Undetected	Very low
Liver	PDI	4	0.83 (0.71, 0.97)	0.0	Serious	Not serious	Not serious	Not serious	Undetected	Moderate
Liver	hPDI	4	0.77 (0.66, 0.90)	0.0	Serious	Not serious	Not serious	Not serious	Undetected	Moderate
Liver	uPDI	4	1.06 (0.92, 1.22)	0.0	Serious	Not serious	Not serious	Serious	Undetected	Low
Lung	PDI/PVG	3	0.76 (0.68, 0.85)	20.9	Serious	Not serious	Not serious	Not serious	Undetected	Moderate

As all included evidence derived from observational studies, certainty started at low. Downgrading decisions were based on predefined criteria: risk of bias was rated as serious when most contributing studies presented concerns in the ROBINS-E domains; inconsistency was considered serious when substantial statistical heterogeneity was observed (I^2 >50%) without a clear explanation; indirectness was considered not serious when population, exposure and outcomes were directly relevant to the review question; imprecision was rated serious when confidence intervals were wide or crossed the null and the total number of cases was limited; publication bias was not downgraded due to the small number of studies per outcome, which precluded reliable assessment. Certainty was upgraded when evidence of dose-response relationships or consistent associations across large prospective cohorts was observed.

Supplementary Table 5. Association between plant-based dietary indices and cancer risk in prospective cohort studies.

Author, year	Cohort name, country/region	Cancer site	Dietary index	Exposure contrast	HR (95% CI)	Included in M-A
Kane-Diallo A et al, 2018	NutriNet Santé cohort, France	Breast	PVG	Highest vs lowest category	0.86 (0.69, 1.08)	Yes
Martínez CF et al, 2023	Moli-Sani Study, Italy	Breast	PDI	Highest vs lowest category	0.94 (0.66, 1.36)	Yes
Martínez CF et al, 2023	Moli-Sani Study, Italy	Breast	PDI	Per 1-SD increase	0.98 (0.87, 1.10)	Yes
Martínez CF et al, 2023	Moli-Sani Study, Italy	Breast	hPDI	Highest vs lowest category	0.98 (0.87, 1.10)	Yes
Martínez CF et al, 2023	Moli-Sani Study, Italy	Breast	hPDI	Per 1-SD increase	0.85 (0.58, 1.24)	Yes
Martínez CF et al, 2023	Moli-Sani Study, Italy	Breast	uPDI	Highest vs lowest category	1.09 (0.96, 1.22)	Yes
Martínez CF et al, 2023	Moli-Sani Study, Italy	Breast	uPDI	Per 1-SD increase	1.40 (0.95, 2.05)	Yes
Romanos-Nanclares A et al, 2020	SUN cohort, Spain	Breast	PDI	Highest vs lowest category	1.03 (0.67, 1.60)	Yes
Romanos-Nanclares A et al, 2020	SUN cohort, Spain	Breast	hPDI	Highest vs lowest category	0.83 (0.50, 1.37)	Yes
Romanos-Nanclares A et al, 2020	SUN cohort, Spain	Breast	uPDI	Highest vs lowest category	1.31 (0.79, 2.19)	Yes
Romanos-Nanclares A et al, 2021	NHS and NHSII, USA	Breast	PDI	Highest vs lowest category	0.89 (0.84, 0.95)	Yes
Romanos-Nanclares A et al, 2021	NHS and NHSII, USA	Breast	PDI	Per 10-unit increase	0.92 (0.89, 0.96)	Yes
Romanos-Nanclares A et al, 2021	NHS and NHSII, USA	Breast	hPDI	Highest vs lowest category	0.89 (0.83, 0.94)	Yes
Romanos-Nanclares A et al, 2021	NHS and NHSII, USA	Breast	hPDI	Per 10-unit increase	0.94 (0.91, 0.97)	Yes
Romanos-Nanclares A et al, 2021	NHS and NHSII, USA	Breast	uPDI	Highest vs lowest category	1.04 (0.97, 1.10)	Yes
Romanos-Nanclares A et al, 2021	NHS and NHSII, USA	Breast	uPDI	Per 10-unit increase	1.02 (0.99, 1.05)	Yes
Shah S et al, 2022	E3N, France	Breast	hPDI	Highest vs lowest category	0.86 (0.77, 0.95)	Yes
Shah S et al, 2022	E3N, France	Breast	uPDI	Highest vs lowest category	1.20 (1.08, 1.33)	Yes
Shah S et al, 2022	E3N, France	Breast	uPDI	Per 1-SD increase	1.04 (1.01, 1.08)	Yes
Shah S et al, 2025	EPIC, Europe	Breast	PDI	Highest vs lowest category	0.95 (0.88, 1.02)	Yes
Shah S et al, 2025	EPIC, Europe	Breast	PDI	Per 1-SD increase	0.98 (0.96, 1.00)	Yes
Shah S et al, 2025	EPIC, Europe	Breast	hPDI	Highest vs lowest category	0.89 (0.83, 0.95)	Yes

Author, year	Cohort name, country/region	Cancer site	Dietary index	Exposure contrast	HR (95% CI)	Included in M-A
Shah S et al, 2025	EPIC, Europe	Breast	hPDI	Per 1-SD increase	0.97 (0.95, 0.99)	Yes
Shah S et al, 2025	EPIC, Europe	Breast	uPDI	Highest vs lowest category	1.02 (0.95, 1.09)	Yes
Shah S et al, 2025	EPIC, Europe	Breast	uPDI	Per 1-SD increase	1.01 (0.99, 1.03)	Yes
Thompson AS et al, 2023	UK Biobank, UK	Breast	hPDI	Highest vs lowest category	0.99 (0.83, 1.19)	Yes
Thompson AS et al, 2023	UK Biobank, UK	Breast	uPDI	Highest vs lowest category	1.16 (0.97, 1.38)	Yes
Kim J et al, 2022	Multiethnic Cohort Study, USA (W)	Colorectum	PDI	Highest vs lowest category	0.99 (0.86, 1.14)	Yes
Kim J et al, 2022	Multiethnic Cohort Study, USA (M)	Colorectum	PDI	Highest vs lowest category	0.76 (0.67, 0.87)	Yes
Kim J et al, 2022	Multiethnic Cohort Study, USA (W)	Colorectum	hPDI	Highest vs lowest category	0.91 (0.80, 1.04)	Yes
Kim J et al, 2022	Multiethnic Cohort Study, USA (M)	Colorectum	hPDI	Highest vs lowest category	0.79 (0.69, 0.91)	Yes
Kim J et al, 2022	Multiethnic Cohort Study, USA (W)	Colorectum	uPDI	Highest vs lowest category	1.01 (0.89, 1.15)	Yes
Kim J et al, 2022	Multiethnic Cohort Study, USA (M)	Colorectum	uPDI	Highest vs lowest category	1.08 (0.95, 1.22)	Yes
Kim J et al, 2023	NHS, NHSII and HPFS, USA	Colorectum	PDI	Per 10-unit increase	0.98 (0.92, 1.05)	Yes
Kim J et al, 2023	NHS, NHSII and HPFS, USA	Colorectum	hPDI	Per 10-unit increase	0.94 (0.89, 1.00)	Yes
Kim J et al, 2023	NHS, NHSII and HPFS, USA	Colorectum	uPDI	Per 10-unit increase	1.07 (1.01, 1.13)	Yes
Liu F et al, 2023	UK Biobank, UK	Colorectum	PDI	Highest vs lowest category	0.87 (0.77, 0.99)	Yes
Liu F et al, 2023	UK Biobank, UK	Colorectum	PDI	Per 10-unit increase	0.88 (0.81, 0.96)	Yes
Liu F et al, 2023	UK Biobank, UK	Colorectum	hPDI	Highest vs lowest category	0.85 (0.75, 0.97)	Yes
Liu F et al, 2023	UK Biobank, UK	Colorectum	hPDI	Per 10-unit increase	0.91 (0.84, 0.99)	Yes
Liu F et al, 2023	UK Biobank, UK	Colorectum	uPDI	Highest vs lowest category	1.14 (1.01, 1.30)	Yes
Liu F et al, 2023	UK Biobank, UK	Colorectum	uPDI	Per 10-unit increase	1.05 (0.97, 1.13)	Yes
Yuan F et al, 2025	SCCS, USA	Colorectum	PDI	Highest vs lowest category	0.87 (0.70, 1.08)	Yes
Yuan F et al, 2025	SCCS, USA	Colorectum	PDI	Per 10-unit increase	0.92 (0.81, 1.05)	Yes
Yuan F et al, 2025	SCCS, USA	Colorectum	hPDI	Highest vs lowest category	1.20 (0.94, 1.53)	Yes

Author, year	Cohort name, country/region	Cancer site	Dietary index	Exposure contrast	HR (95% CI)	Included in M-A
Yuan F et al, 2025	SCCS, USA	Colorectum	hPDI	Per 10-unit increase	1.03 (0.91, 1.17)	Yes
Yuan F et al, 2025	SCCS, USA	Colorectum	uPDI	Highest vs lowest category	1.21 (0.96, 1.52)	Yes
Yuan F et al, 2025	SCCS, USA	Colorectum	uPDI	Per 10-unit increase	1.10 (0.98, 1.23)	Yes
Kane-Diallo A et al, 2018	NutriNet Santé cohort, France	Prostate	PVG	Highest vs lowest category	0.76 (0.55, 1.06)	Yes
Loeb S et al, 2022	HPFS, USA	Prostate	PDI	Highest vs lowest category	0.97 (0.90, 1.05)	Yes
Loeb S et al, 2022	HPFS, USA	Prostate	hPDI	Highest vs lowest category	0.95 (0.87, 1.02)	Yes
Martínez CF et al, 2023	Moli-Sani Study, Italy	Prostate	PDI	Highest vs lowest category	1.08 (0.94, 1.24)	Yes
Martínez CF et al, 2023	Moli-Sani Study, Italy	Prostate	PDI	Per 1-SD increase	1.37 (0.88, 2.13)	Yes
Martínez CF et al, 2023	Moli-Sani Study, Italy	Prostate	hPDI	Highest vs lowest category	1.07 (0.94, 1.23)	Yes
Martínez CF et al, 2023	Moli-Sani Study, Italy	Prostate	hPDI	Per 1-SD increase	1.37 (0.84, 2.23)	No
Martínez CF et al, 2023	Moli-Sani Study, Italy	Prostate	uPDI	Highest vs lowest category	1.03 (0.90, 1.19)	No
Martínez CF et al, 2023	Moli-Sani Study, Italy	Prostate	uPDI	Per 1-SD increase	1.21 (0.80, 1.82)	No
Thompson AS et al, 2023	UK Biobank, UK	Prostate	hPDI	Highest vs lowest category	0.98 (0.86, 1.11)	Yes
Thompson AS et al, 2023	UK Biobank, UK	Prostate	uPDI	Highest vs lowest category	1.05 (0.93, 1.19)	No
Kane-Diallo A et al, 2018	NutriNet Santé cohort, France	Lung	PVG	Highest vs lowest category	0.47 (0.24, 0.90)	Yes
Wei W et al, 2025	PLCO Cancer Screening Trial, USA	Lung	PDI	Highest vs lowest category	0.75 (0.65, 0.87)	Yes
Zhu W et al, 2025	UK Biobank, UK	Lung	PDI	Highest vs lowest category	0.81 (0.68, 0.98)	Yes
Zhu W et al, 2025	UK Biobank, UK	Lung	PDI	Per 1-SD increase	0.90 (0.85, 0.96)	Yes
Zhu W et al, 2025	UK Biobank, UK	Lung	hPDI	Highest vs lowest category	1.02 (0.85, 1.24)	No
Zhu W et al, 2025	UK Biobank, UK	Lung	hPDI	Per 1-SD increase	1.01 (0.95, 1.08)	No
Zhu W et al, 2025	UK Biobank, UK	Lung	uPDI	Highest vs lowest category	1.25 (1.05, 1.48)	No
Zhu W et al, 2025	UK Biobank, UK	Lung	uPDI	Per 1-SD increase	1.08 (1.01, 1.15)	No
Dong X et al, 2025	UK Biobank, UK (W)	Liver	PDI	Highest vs lowest category	0.67 (0.35, 1.26)	Yes

Author, year	Cohort name, country/region	Cancer site	Dietary index	Exposure contrast	HR (95% CI)	Included in M-A
Dong X et al, 2026	UK Biobank, UK (M)	Liver	PDI	Highest vs lowest category	1.42 (0.65, 3.10)	Yes
Dong X et al, 2027	UK Biobank, UK (W)	Liver	hPDI	Highest vs lowest category	0.47 (0.26, 0.85)	Yes
Dong X et al, 2028	UK Biobank, UK (M)	Liver	hPDI	Highest vs lowest category	1.92 (0.76, 4.82)	Yes
Dong X et al, 2029	UK Biobank, UK (W)	Liver	uPDI	Highest vs lowest category	1.90 (1.00, 3.63)	Yes
Dong X et al, 2030	UK Biobank, UK (M)	Liver	uPDI	Highest vs lowest category	0.78 (0.36, 1.67)	Yes
Kim J et al, 2023	NHS, NHSII and HPFS, USA	Liver	PDI	Per 10-unit increase	0.69 (0.50, 0.95)	Yes
Kim J et al, 2023	NHS, NHSII and HPFS, USA	Liver	hPDI	Per 10-unit increase	0.68 (0.52, 0.91)	Yes
Kim J et al, 2023	NHS, NHSII and HPFS, USA	Liver	uPDI	Per 10-unit increase	1.08 (0.83, 1.40)	Yes
Yuan F et al, 2025	SCCS, USA	Liver	PDI	Highest vs lowest category	0.89 (0.65, 1.22)	Yes
Yuan F et al, 2025	SCCS, USA	Liver	PDI	Per 10-unit increase	0.86 (0.71, 1.04)	Yes
Yuan F et al, 2025	SCCS, USA	Liver	hPDI	Highest vs lowest category	0.67 (0.45, 0.99)	Yes
Yuan F et al, 2025	SCCS, USA	Liver	hPDI	Per 10-unit increase	0.80 (0.66, 0.98)	Yes
Yuan F et al, 2025	SCCS, USA	Liver	uPDI	Highest vs lowest category	1.35 (0.95, 1.93)	Yes
Yuan F et al, 2025	SCCS, USA	Liver	uPDI	Per 10-unit increase	1.06 (0.89, 1.26)	Yes
Kim J et al, 2023	Multiethnic Cohort Study, USA	Hepatocellular carcinoma	PDI	Highest vs lowest category	0.77 (0.61, 0.98)	No
Kim J et al, 2023	Multiethnic Cohort Study, USA	Hepatocellular carcinoma	PDI	Per 10-unit increase	0.82 (0.71, 0.94)	No
Kim J et al, 2023	Multiethnic Cohort Study, USA	Hepatocellular carcinoma	hPDI	Highest vs lowest category	0.81 (0.63, 1.03)	No
Kim J et al, 2023	Multiethnic Cohort Study, USA	Hepatocellular carcinoma	hPDI	Per 10-unit increase	0.84 (0.74, 0.96)	No
Kim J et al, 2023	Multiethnic Cohort Study, USA	Hepatocellular carcinoma	uPDI	Highest vs lowest category	1.13 (0.88, 1.44)	No
Kim J et al, 2023	Multiethnic Cohort Study, USA	Hepatocellular carcinoma	uPDI	Per 10-unit increase	1.08 (0.95, 1.23)	No
Pham Y et al, 2025	SCHS, Singapur	Hepatocellular carcinoma	PDI	Highest vs lowest category	0.73 (0.56, 0.93)	No
Pham Y et al, 2025	SCHS, Singapur	Hepatocellular carcinoma	PDI	Per 1-SD increase	0.87(0.79, 0.95)	No
Pham Y et al, 2025	SCHS, Singapur	Hepatocellular carcinoma	hPDI	Highest vs lowest category	0.69 (0.53, 0.89)	No

Author, year	Cohort name, country/region	Cancer site	Dietary index	Exposure contrast	HR (95% CI)	Included in M-A
Pham Y et al, 2025	SCHS, Singapur	Hepatocellular carcinoma	hPDI	Per 1-SD increase	0.86 (0.79, 0.94)	No
Pham Y et al, 2025	SCHS, Singapur	Hepatocellular carcinoma	uPDI	Highest vs lowest category	1.22 (0.93, 1.59)	No
Pham Y et al, 2025	SCHS, Singapur	Hepatocellular carcinoma	uPDI	Per 1-SD increase	1.06 (0.96, 1.17)	No
Kim J et al, 2023	NHS, NHSII and HPFS, USA	Pancreas	PDI	Per 10-unit increase	0.83 (0.73, 0.94)	No
Kim J et al, 2023	NHS, NHSII and HPFS, USA	Pancreas	hPDI	Per 10-unit increase	0.91 (0.81, 1.01)	No
Kim J et al, 2023	NHS, NHSII and HPFS, USA	Pancreas	uPDI	Per 10-unit increase	0.93 (0.84, 1.04)	No
Zhong G et al, 2023	PLCO Cancer Screening Trial, USA	Pancreas	PDI	Highest vs lowest category	0.74 (0.57, 0.96)	No
Zhong G et al, 2023	PLCO Cancer Screening Trial, USA	Pancreas	hPDI	Highest vs lowest category	0.56 (0.42, 0.75)	No
Zhong G et al, 2023	PLCO Cancer Screening Trial, USA	Pancreas	uPDI	Highest vs lowest category	1.38 (1.02, 1.85)	No
Thi-Hai Pham Y et al, 2025	SCHS, Singapur	Pancreas	PDI	Highest vs lowest category	1.02 (0.72, 1.46)	No
Thi-Hai Pham Y et al, 2025	SCHS, Singapur	Pancreas	PDI	Per 1-SD increase	0.99 (0.88, 1.12)	No
Thi-Hai Pham Y et al, 2025	SCHS, Singapur	Pancreas	hPDI	Highest vs lowest category	0.84 (0.59, 1.18)	No
Thi-Hai Pham Y et al, 2025	SCHS, Singapur	Pancreas	hPDI	Per 1-SD increase	0.90 (0.80, 1.01)	No
Thi-Hai Pham Y et al, 2025	SCHS, Singapur	Pancreas	uPDI	Highest vs lowest category	1.68 (1.18, 2.39)	No
Thi-Hai Pham Y et al, 2025	SCHS, Singapur	Pancreas	uPDI	Per 1-SD increase	1.20 (1.06, 1.37)	No
Kane-Diallo A et al, 2018	NutriNet Santé cohort, France	Digestive organs	PVG	Highest vs lowest category	0.68 (0.47, 0.99)	No
Martínez CF et al, 2023	Moli-Sani Study, Italy	Digestive organs	PDI	Highest vs lowest category	0.88 (0.81, 0.96)	No
Martínez CF et al, 2023	Moli-Sani Study, Italy	Digestive organs	PDI	Per 1-SD increase	0.74 (0.57, 0.95)	No
Martínez CF et al, 2023	Moli-Sani Study, Italy	Digestive organs	hPDI	Highest vs lowest category	0.92 (0.85, 1.00)	No
Martínez CF et al, 2023	Moli-Sani Study, Italy	Digestive organs	hPDI	Per 1-SD increase	0.76 (0.58, 0.99)	No
Martínez CF et al, 2023	Moli-Sani Study, Italy	Digestive organs	uPDI	Highest vs lowest category	1.00 (0.92, 1.08)	No
Martínez CF et al, 2023	Moli-Sani Study, Italy	Digestive organs	uPDI	Per 1-SD increase	0.92 (0.70, 1.21)	No
Kim J et al, 2023	NHS, NHSII and HPFS, USA	Oral cavity/oropharynge	PDI	Per 10-unit increase	0.78 (0.65, 0.95)	No

Author, year	Cohort name, country/region	Cancer site	Dietary index	Exposure contrast	HR (95% CI)	Included in M-A
Kim J et al, 2023	NHS, NHSII and HPFS, USA	Oral cavity/oropharynge	hPDI	Per 10-unit increase	0.86 (0.73, 1.02)	No
Kim J et al, 2023	NHS, NHSII and HPFS, USA	Oral cavity/oropharynge	uPDI	Per 10-unit increase	0.97 (0.83, 1.13)	No
Kim J et al, 2023	NHS, NHSII and HPFS, USA	Small intestine	PDI	Per 10-unit increase	0.86 (0.60, 1.24)	No
Kim J et al, 2023	NHS, NHSII and HPFS, USA	Small intestine	hPDI	Per 10-unit increase	0.86 (0.63, 1.18)	No
Kim J et al, 2023	NHS, NHSII and HPFS, USA	Small intestine	uPDI	Per 10-unit increase	0.87 (0.65, 1.17)	No
Kim J et al, 2023	NHS, NHSII and HPFS, USA	Esophagus	PDI	Per 10-unit increase	1.09 (0.86, 1.40)	No
Kim J et al, 2023	NHS, NHSII and HPFS, USA	Esophagus	hPDI	Per 10-unit increase	0.91 (0.74, 1.12)	No
Kim J et al, 2023	NHS, NHSII and HPFS, USA	Esophagus	uPDI	Per 10-unit increase	1.12 (0.91, 1.37)	No
Kim J et al, 2023	NHS, NHSII and HPFS, USA	Colon	PDI	Per 10-unit increase	0.98 (0.90, 1.07)	No
Kim J et al, 2023	NHS, NHSII and HPFS, USA	Colon	hPDI	Per 10-unit increase	0.94 (0.87, 1.01)	No
Kim J et al, 2023	NHS, NHSII and HPFS, USA	Colon	uPDI	Per 10-unit increase	1.04 (0.97, 1.11)	No
Kim J et al, 2023	NHS, NHSII and HPFS, USA	Rectum	PDI	Per 10-unit increase	1.02 (0.88, 1.19)	No
Kim J et al, 2023	NHS, NHSII and HPFS, USA	Rectum	hPDI	Per 10-unit increase	0.99 (0.87, 1.13)	No
Kim J et al, 2023	NHS, NHSII and HPFS, USA	Rectum	uPDI	Per 10-unit increase	1.08 (0.96, 1.22)	No
Kim J et al, 2023	NHS, NHSII and HPFS, USA	Accessory organ	PDI	Per 10-unit increase	0.84 (0.75, 0.94)	No
Kim J et al, 2023	NHS, NHSII and HPFS, USA	Accessory organ	hPDI	Per 10-unit increase	0.89 (0.81, 0.98)	No
Kim J et al, 2023	NHS, NHSII and HPFS, USA	Accessory organ	uPDI	Per 10-unit increase	0.97 (0.89, 1.06)	No
Kim J et al, 2023	NHS, NHSII and HPFS, USA	Biliary tract	PDI	Per 10-unit increase	1.01 (0.77, 1.32)	No
Kim J et al, 2023	NHS, NHSII and HPFS, USA	Biliary tract	hPDI	Per 10-unit increase	0.98 (0.78, 1.23)	No
Kim J et al, 2023	NHS, NHSII and HPFS, USA	Biliary tract	uPDI	Per 10-unit increase	1.04 (0.84, 1.29)	No
Martínez CF et al, 2023	Moli-Sani Study, Italy	Respiratory tract	PDI	Highest vs lowest category	0.88 (0.76, 1.03)	No
Martínez CF et al, 2023	Moli-Sani Study, Italy	Respiratory tract	PDI	Per 1-SD increase	0.61 (0.38, 0.98)	No
Martínez CF et al, 2023	Moli-Sani Study, Italy	Respiratory tract	hPDI	Highest vs lowest category	0.87 (0.75, 1.02)	No

Author, year	Cohort name, country/region	Cancer site	Dietary index	Exposure contrast	HR (95% CI)	Included in M-A
Martínez CF et al, 2023	Moli-Sani Study, Italy	Respiratory tract	hPDI	Per 1-SD increase	0.70 (0.41, 1.19)	No
Martínez CF et al, 2023	Moli-Sani Study, Italy	Respiratory tract	uPDI	Highest vs lowest category	1.14 (0.98, 1.33)	No
Martínez CF et al, 2023	Moli-Sani Study, Italy	Respiratory tract	uPDI	Per 1-SD increase	1.68 (1.06, 2.68)	No
Martínez CF et al, 2023	Moli-Sani Study, Italy	Genitourinary organs	PDI	Highest vs lowest category	0.97 (0.87, 1.08)	No
Martínez CF et al, 2023	Moli-Sani Study, Italy	Genitourinary organs	PDI	Per 1-SD increase	0.86 (0.63, 1.18)	No
Martínez CF et al, 2023	Moli-Sani Study, Italy	Genitourinary organs	hPDI	Highest vs lowest category	1.00 (0.90, 1.11)	No
Martínez CF et al, 2023	Moli-Sani Study, Italy	Genitourinary organs	hPDI	Per 1-SD increase	1.07 (0.76, 1.51)	No
Martínez CF et al, 2023	Moli-Sani Study, Italy	Genitourinary organs	uPDI	Highest vs lowest category	0.99 (0.90, 1.10)	No
Martínez CF et al, 2023	Moli-Sani Study, Italy	Genitourinary organs	uPDI	Per 1-SD increase	0.94 (0.68, 1.29)	No
Martínez CF et al, 2023	Moli-Sani Study, Italy	Lymphatic, hematopoietic	PDI	Highest vs lowest category	1.03 (0.88, 1.20)	No
Martínez CF et al, 2023	Moli-Sani Study, Italy	Lymphatic, hematopoietic	PDI	Per 1-SD increase	1.03 (0.65, 1.63)	No
Martínez CF et al, 2023	Moli-Sani Study, Italy	Lymphatic, hematopoietic	hPDI	Highest vs lowest category	1.06 (0.90, 1.23)	No
Martínez CF et al, 2023	Moli-Sani Study, Italy	Lymphatic, hematopoietic	hPDI	Per 1-SD increase	1.03 (0.63, 1.71)	No
Martínez CF et al, 2023	Moli-Sani Study, Italy	Lymphatic, hematopoietic	uPDI	Highest vs lowest category	1.04 (0.90, 1.22)	No
Martínez CF et al, 2023	Moli-Sani Study, Italy	Lymphatic, hematopoietic	uPDI	Per 1-SD increase	1.24 (0.77, 2.00)	No
Martínez CF et al, 2023	Moli-Sani Study, Italy	Brain and nervous system	PDI	Highest vs lowest category	0.93 (0.68, 1.27)	No
Martínez CF et al, 2023	Moli-Sani Study, Italy	Brain and nervous system	PDI	Per 1-SD increase	0.92 (0.37, 2.29)	No
Martínez CF et al, 2023	Moli-Sani Study, Italy	Brain and nervous system	hPDI	Highest vs lowest category	1.15 (0.85, 1.56)	No
Martínez CF et al, 2023	Moli-Sani Study, Italy	Brain and nervous system	hPDI	Per 1-SD increase	1.36 (0.54, 3.44)	No
Martínez CF et al, 2023	Moli-Sani Study, Italy	Brain and nervous system	uPDI	Highest vs lowest category	1.04 (0.76, 1.43)	No
Martínez CF et al, 2023	Moli-Sani Study, Italy	Brain and nervous system	uPDI	Per 1-SD increase	0.82 (0.31, 2.18)	No

CI: confidence interval, HR: hazard ratio, M: men, UK: United Kingdom; USA: United States of America, W: women. Study names: E3N: Étude Épidémiologique auprès de femmes de la Mutuelle Générale de l'Éducation Nationale, EPIC: European Prospective Investigation into Cancer and Nutrition, HPFS: Health Professionals Follow-up Study, NHS: Nurses' Health Study, PLCO: Prostate, Lung, Colorectal, and

Ovarian Cancer Screening Trial, SCCS: Southern Community Cohort Study; SCHS: Singapore Chinese Health Study; SUN: Seguimiento Universidad de Navarra. Dietary indices: PDI: plant-based diet index, hPDI: healthful plant-based diet index, uPDI: unhealthful plant-based diet index, PVG: pro-vegetarian pattern. HR and 95% CI correspond to the most fully-adjusted model. Covariate adjustment was generally comprehensive, including age, sex, body mass index, smoking status, education, physical activity, and total energy intake.

Supplementary Table 6. Results of leave-one-out sensitivity analyses assessing the robustness of meta-analytic estimates.

Cancer site	Dietary index	Contrast	Omitted study	K	Pooled HR (95% CI)	I^2 (%)	Significance retained	τ^2
Breast	PDI	Per 10-unit increase	Overall (all studies)	5	0.93 (0.88, 0.99)	65.2	Yes	<0.01
Breast	PDI	Per 10-unit increase	Kane-Diallo A et al, 2018. NutriNet Santé cohort, France	4	0.96 (0.91, 1.00)	71.5	No	<0.01
Breast	PDI	Per 10-unit increase	Martínez CF et al, 2023. Moli-Sani Study, Italy	4	0.92 (0.86, 0.99)	80.4	Yes	<0.01
Breast	PDI	Per 10-unit increase	Romanos-Nanclares A et al, 2020. SUN cohort, Spain	4	0.93 (0.87, 0.99)	85.9	Yes	<0.01
Breast	PDI	Per 10-unit increase	Romanos-Nanclares A et al, 2021. NHS and NHSII, USA	4	0.94 (0.85, 1.02)	49.8	No	<0.01
Breast	PDI	Per 10-unit increase	Shah S et al, 2025. EPIC, Europe	4	0.91 (0.85, 0.97)	0.0	Yes	<0.01
Breast	hPDI	Per 10-unit increase	Overall (all studies)	5	0.94 (0.92, 0.97)	79.4	Yes	<0.01
Breast	hPDI	Per 10-unit increase	Martínez CF et al, 2023. Moli-Sani Study, Italy	4	0.94 (0.92, 0.97)	64.5	Yes	<0.01
Breast	hPDI	Per 10-unit increase	Romanos-Nanclares A et al, 2020. SUN cohort, Spain	4	0.93 (0.88, 0.99)	86.4	Yes	<0.01
Breast	hPDI	Per 10-unit increase	Romanos-Nanclares A et al, 2021. NHS and NHSII, USA	4	0.93 (0.88, 0.99)	84.6	Yes	<0.01
Breast	hPDI	Per 10-unit increase	Shah S et al, 2023. E3N, France	4	0.95 (0.93, 0.97)	70.3	Yes	<0.01
Breast	hPDI	Per 10-unit increase	Shah S et al, 2025. EPIC, Europe	4	0.94 (0.92, 0.95)	43.8	Yes	<0.01
Breast	uPDI	Per 10-unit increase	Overall (all studies)	5	1.02 (1.01, 1.04)	24.0	Yes	<0.01
Breast	uPDI	Per 10-unit increase	Martínez CF et al, 2023. Moli-Sani Study, Italy	4	1.02 (1.00, 1.04)	25.1	Yes	<0.01
Breast	uPDI	Per 10-unit increase	Romanos-Nanclares A et al, 2020. SUN cohort, Spain	4	1.02 (1.00, 1.04)	9.3	Yes	<0.01
Breast	uPDI	Per 10-unit increase	Romanos-Nanclares A et al, 2021. NHS and NHSII, USA	4	1.04 (1.00, 1.09)	68.1	No	<0.01
Breast	uPDI	Per 10-unit increase	Shah S et al, 2023. E3N, France	4	1.02 (1.00, 1.03)	0.6	No	<0.01
Breast	uPDI	Per 10-unit increase	Shah S et al, 2025. EPIC, Europe	4	1.04 (1.01, 1.06)	10.4	Yes	<0.01
Colorectum	PDI	Per 10-unit increase	Overall (all studies)	5	0.95 (0.91, 0.98)	56.0	Yes	<0.01
Colorectum	PDI	Per 10-unit increase	Kim J et al, 2022. Multiethnic Cohort Study, USA (W)	4	0.93 (0.90, 0.96)	0.5	Yes	<0.01
Colorectum	PDI	Per 10-unit increase	Kim J et al, 2022. Multiethnic Cohort Study, USA (M)	4	0.95 (0.90, 1.00)	31.9	No	<0.01

Cancer site	Dietary index	Contrast	Omitted study	K	Pooled HR (95% CI)	I^2 (%)	Significance retained	τ^2
Colorectum	PDI	Per 10-unit increase	Kim J et al, 2023. NHS, NHSII and HPFS, USA	4	0.94 (0.90, 0.98)	68.0	Yes	<0.01
Colorectum	PDI	Per 10-unit increase	Liu F et al, 2023. UK Biobank, UK	4	0.96 (0.92, 0.99)	63.6	Yes	<0.01
Colorectum	PDI	Per 10-unit increase	Yuan F et al, 2025. SCCS, USA	4	0.95 (0.91, 0.99)	61.6	Yes	<0.01
Colorectum	hPDI	Per 10-unit increase	Overall (all studies)	5	0.95 (0.92, 0.98)	62.1	Yes	<0.01
Colorectum	hPDI	Per 10-unit increase	Kim J et al, 2022. Multiethnic Cohort Study, USA (W)	4	0.93 (0.91, 0.95)	18.4	Yes	<0.01
Colorectum	hPDI	Per 10-unit increase	Kim J et al, 2022. Multiethnic Cohort Study, USA (M)	4	0.96 (0.93, 1.00)	55.8	Yes	<0.01
Colorectum	hPDI	Per 10-unit increase	Kim J et al, 2023. NHS, NHSII and HPFS, USA	4	0.95 (0.92, 0.99)	71.2	Yes	<0.01
Colorectum	hPDI	Per 10-unit increase	Liu F et al, 2023. UK Biobank, UK	4	0.96 (0.92, 0.99)	57.4	Yes	<0.01
Colorectum	hPDI	Per 10-unit increase	Yuan F et al, 2025. SCCS, USA	4	0.95 (0.92, 0.98)	70.9	Yes	<0.01
Colorectum	uPDI	Per 10-unit increase	Overall (all studies)	5	1.03 (0.99, 1.06)	70.5	No	<0.01
Colorectum	uPDI	Per 10-unit increase	Kim J et al, 2022. Multiethnic Cohort Study, USA (W)	4	1.04 (1.00, 1.09)	49.9	No	<0.01
Colorectum	uPDI	Per 10-unit increase	Kim J et al, 2022. Multiethnic Cohort Study, USA (M)	4	1.04 (0.99, 1.09)	68.5	No	<0.01
Colorectum	uPDI	Per 10-unit increase	Kim J et al, 2023. NHS, NHSII and HPFS, USA	4	1.00 (0.98, 1.03)	31.7	No	<0.01
Colorectum	uPDI	Per 10-unit increase	Liu F et al, 2023. UK Biobank, UK	4	1.02 (0.98, 1.07)	77.2	No	<0.01
Colorectum	uPDI	Per 10-unit increase	Yuan F et al, 2025. SCCS, USA	4	1.02 (0.98, 1.06)	71.1	No	<0.01
Prostate	PDI	Per 10-unit increase	Overall (all studies)	3	0.94 (0.73, 1.23)	97.4	No	0.04
Prostate	PDI	Per 10-unit increase	Kane-Diallo A et al, 2018. NutriNet Santé cohort, France	2	1.15 (0.72, 1.84)	48.4	No	0.08
Prostate	PDI	Per 10-unit increase	Loeb S et al, 2022. HPFS, USA.	2	1.06 (0.50, 2.26)	74.4	No	0.24
Prostate	PDI	Per 10-unit increase	Martínez CF et al, 2023. Moli-Sani Study, Italy	2	0.89 (0.71, 1.12)	98.1	No	0.03
Liver	PDI	Per 10-unit increase	Overall (all studies)	4	0.83 (0.71, 0.97)	0.0	Yes	0.00
Liver	PDI	Per 10-unit increase	Dong X et al, 2025. UK Biobank, UK (W)	3	0.80 (0.67, 0.96)	0.0	Yes	<0.01
Liver	PDI	Per 10-unit increase	Dong X et al, 2025. UK Biobank, UK (M)	3	0.83 (0.71, 0.98)	0.0	Yes	<0.01

Cancer site	Dietary index	Contrast	Omitted study	K	Pooled HR (95% CI)	I^2 (%)	Significance retained	τ^2
Liver	PDI	Per 10-unit increase	Kim J et al, 2023. NHS, NHSII and HPFS, USA	3	0.88 (0.73, 1.05)	0.0	No	0.00
Liver	PDI	Per 10-unit increase	Yuan F et al, 2025. SCCS, USA	3	0.79 (0.57, 1.11)	21.7	No	<0.01
Liver	hPDI	Per 10-unit increase	Overall (all studies)	4	0.77 (0.66, 0.91)	0.0	Yes	0.00
Liver	hPDI	Per 10-unit increase	Dong X et al, 2025. UK Biobank, UK (W)	3	0.76 (0.65, 0.89)	9.9	Yes	0.00
Liver	hPDI	Per 10-unit increase	Dong X et al, 2025. UK Biobank, UK (M)	3	0.77 (0.66, 0.90)	2.3	Yes	0.00
Liver	hPDI	Per 10-unit increase	Kim J et al, 2023. NHS, NHSII and HPFS, USA	3	0.82 (0.68, 0.99)	0.0	Yes	0.00
Liver	hPDI	Per 10-unit increase	Yuan F et al, 2025. SCCS, USA	3	0.79 (0.52, 1.20)	20.1	No	0.04
Liver	uPDI	Per 10-unit increase	Overall (all studies)	4	1.06 (0.92, 1.22)	0.0	No	0.00
Liver	uPDI	Per 10-unit increase	Dong X et al, 2025. UK Biobank, UK (W)	3	1.07 (0.93, 1.24)	0.0	No	0.00
Liver	uPDI	Per 10-unit increase	Dong X et al, 2025. UK Biobank, UK (M)	3	1.06 (0.92, 1.21)	0.0	No	0.00
Liver	uPDI	Per 10-unit increase	Kim J et al, 2023. NHS, NHSII and HPFS, USA	3	1.05 (0.90, 1.24)	0.0	No	0.00
Liver	uPDI	Per 10-unit increase	Yuan F et al, 2025. SCCS, USA	3	1.06 (0.85, 1.33)	0.0	No	0.00
Lung	PDI	Highest vs lowest category	Overall (all studies)	3	0.76 (0.68, 0.85)	0.0	Yes	0.00
Lung	PDI	Highest vs lowest category	Kane-Diallo A et al, 2018. NutriNet Santé cohort, France	2	0.77 (0.69, 0.87)	0.0	Yes	0.00
Lung	PDI	Highest vs lowest category	Wei W et al, 2025. PLCO Cancer Screening Trial, USA	2	0.68 (0.41, 1.12)	58.7	No	0.09
Lung	PDI	Highest vs lowest category	Zhu W et al, 2025. UK Biobank, UK	2	0.67 (0.45, 0.99)	45.4	Yes	0.05

CI: confidence interval, HR: hazard ratio, K: number of studies included in the pooled analysis, M: men, UK: United Kingdom, USA: United States of America, W: women. Study names: E3N: Étude Épidémiologique auprès de femmes de la Mutuelle Générale de l'Éducation Nationale, EPIC: European Prospective Investigation into Cancer and Nutrition, HPFS: Health Professionals Follow-up Study, NHS: Nurses' Health Study, PLCO: Prostate, Lung, Colorectal, and Ovarian Cancer Screening Trial, SCCS: Southern Community Cohort Study, SCHS: Singapore Chinese Health Study, SUN: Seguimiento Universidad de Navarra. Dietary indices: PDI: plant-based diet index, hPDI: healthful plant-based diet index, uPDI: unhealthful plant-based diet index, PVG: pro-vegetarian pattern. For comparability across studies, pooled estimates are presented per 10-unit increase in the dietary index when data allowed. For lung cancer, meta-analysis was conducted using the highest versus lowest category contrast because insufficient data were available to standardize estimates to a per 10-unit increase. PDI and PVG were considered equivalent exposures due to their comparable construct and scoring. I^2 represents the percentage of total variation across studies attributable to between-study heterogeneity; values >50% indicate substantial heterogeneity. τ^2 represents the estimate of between-study variance in the random-effects model.

Supplementary Figure 1. Quality of included cohort studies (n=19) using the ROBINS-E tool.

Study	D1	D2	D3	D4	D5	D6	D7	Overall
Dong X et al, 2025. UK Biobank, UK	−	−	+	−	+	+	+	Moderate
Kane-Diallo A et al, 2018. NutriNet Santé cohort, France	−	+	+	+	+	+	+	Moderate
Kim J et al, 2022. Multiethnic Cohort Study, USA	−	+	+	−	+	+	+	Moderate
Kim J et al, 2023. Multiethnic Cohort Study, USA	−	+	+	−	+	+	+	Moderate
Kim J et al, 2023. NHS, NHSII and HPFS, USA	−	+	+	+	+	+	+	Moderate
Liu F et al, 2023. UK Biobank, UK	−	−	+	−	+	+	+	Moderate
Loeb S et al, 2022. HPFS, USA.	−	+	−	+	+	+	+	Moderate
Martínez CF et al, 2023. Moli-Sani Study, Italy	−	+	+	−	+	+	+	Moderate
Pham Y et al, 2025. SCHS, Singapur	−	+	+	−	+	+	+	Moderate
Romanos-Nanclares A et al, 2020. SUN cohort, Spain	−	+	−	−	+	+	+	Moderate
Romanos-Nanclares A et al, 2021. NHS and NHSII, USA	−	+	+	+	+	+	+	Moderate
Shah S et al, 2023. E3N, France	−	+	+	+	+	+	+	Moderate
Shah S et al, 2025. EPIC, Europe	−	−	+	−	+	+	+	Moderate
Thi-Hai Pham Y et al, 2025. SCHS, Singapore	−	+	+	−	+	+	+	Moderate
Thompson AS et al, 2023. UK Biobank, UK	−	−	+	−	+	+	+	Moderate
Wei W et al, 2025. PLCO Cancer Screening Trial, USA	−	+	−	−	+	+	+	Moderate
Yuan F et al, 2025. SCCS, USA	−	−	+	−	+	+	+	Moderate
Zhong G et al, 2023. PLCO Cancer Screening Trial, USA	−	+	−	−	+	+	+	Moderate
Zhu W et al, 2025. UK Biobank, UK	−	−	+	−	+	+	+	Moderate

Domains: D1: Bias due to confounding; D2: Bias arising from measurement of the exposure; D3: Bias in selection of participants into the study; D4: Bias due to post-exposure interventions; D5: Bias due to missing data; D6: Bias arising from measurement of the outcome; D7: Bias in selection of the reported result.

Judgment: + Low risk of bias − Some concerns

D: domains, E3N: Étude Épidémiologique auprès de femmes de la Mutuelle Générale de l'Éducation Nationale, EPIC: European Prospective Investigation into Cancer and Nutrition, HPFS: Health Professionals Follow-up Study, NHS: Nurses' Health Study, PLCO: Prostate, Lung, Colorectal, and Ovarian Cancer Screening Trial, SCCS: Southern Community Cohort Study; SCHS: Singapore Chinese Health Study; SUN: Seguimiento Universidad de Navarra, UK: United Kingdom, USA: United States of America

Supplementary Figure 2. Meta-analysis of cohort studies assessing plant-based/pro-vegetarian dietary index (PDI/PVG) and breast cancer risk: forest plot showing pooled hazard ratios for the highest versus lowest index category.

Study	logHR	SE(logHR)	Weight (%)	Hazard Ratio	HR	95%-CI
Kane-Diallo A et al, 2018. French NutriNet Santé cohort, France	-0.1508	0.1143	5.6		0.86	[0.69; 1.08]
Martínez CF et al, 2023. Moli-Sani Study, Italy	-0.0619	0.1844	2.2		0.94	[0.65; 1.35]
Romanos-Nanclares A et al, 2020. SUN cohort, Spain	0.0296	0.2221	1.5		1.03	[0.67; 1.59]
Romanos-Nanclares A et al, 2021. NHS and NHSII, USA	-0.1165	0.0314	51.2		0.89	[0.84; 0.95]
Shah S et al, 2025. EPIC, Europe	-0.0513	0.0377	39.6		0.95	[0.88; 1.02]
**Common effect model**			**.**		**0.91**	**[0.87; 0.96]**
**Random effects model**			**100.0**		**0.91**	**[0.87; 0.97]**

Heterogeneity: $I^2 = 0.0\%$, $\tau^2 = 0.0005$, $p = 0.6687$

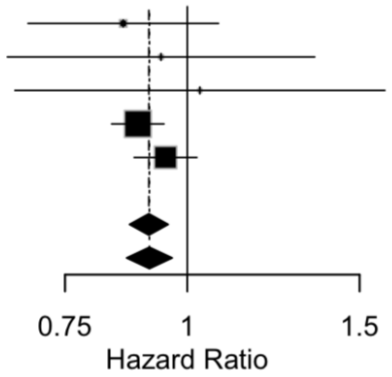

CI: confidence interval, HR: hazard ratio, SE: standard error, USA: United States of America. Study names: EPIC: European Prospective Investigation into Cancer and Nutrition, NHS: Nurses' Health Study, SUN: Seguimiento Universidad de Navarra. I^2 represents the percentage of total variation across studies attributable to between-study heterogeneity; values >50% indicate substantial heterogeneity. τ^2 represents the estimate of between-study variance in the random-effects model. PDI and PVG were considered equivalent exposures due to their comparable construct and scoring.

Supplementary Figure 3. Meta-analysis of cohort studies assessing healthful plant-based dietary index (hPDI) and breast cancer risk: forest plot showing pooled hazard ratios for the highest versus lowest index category.

Study	logHR	SE(logHR)	Weight (%)	HR	95%-CI
Martínez CF et al, 2023. Moli-Sani Study, Italy	-0.0202	0.0598	10.8	0.98	[0.87; 1.10]
Romanos-Nanclares A et al, 2020. SUN cohort, Spain	-0.1863	0.2571	0.6	0.83	[0.50; 1.37]
Romanos-Nanclares A et al, 2021. NHS and NHSII, USA	-0.1165	0.0317	38.2	0.89	[0.84; 0.95]
Shah S et al, 2022. E3N, France	-0.1508	0.0536	13.4	0.86	[0.77; 0.96]
Shah S et al, 2025. EPIC, Europe	-0.1165	0.0344	32.5	0.89	[0.83; 0.95]
Thompson AS et al, 2023. UK Biobank, UK	-0.0101	0.0919	4.6	0.99	[0.83; 1.19]
**Common effect model**			.	**0.90**	**[0.87; 0.93]**
**Random effects model**			**100.0**	**0.90**	**[0.87; 0.93]**

Heterogeneity: $I^2 = 0.0\%$, $\tau^2 = 0$, $p = 0.5286$

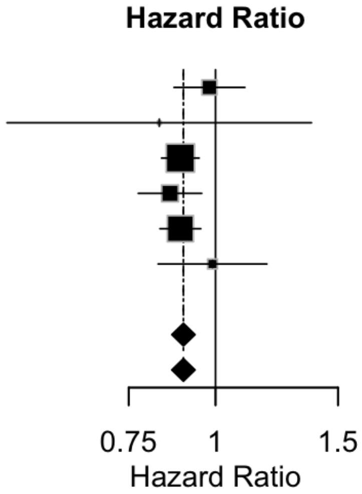

CI: confidence interval, HR: hazard ratio, SE: standard error, UK: United Kingdom, USA: United States of America. Study names: E3N: Étude Épidémiologique auprès de femmes de la Mutuelle Générale de l'Éducation Nationale, EPIC: European Prospective Investigation into Cancer and Nutrition, NHS: Nurses’ Health Study. I² represents the percentage of total variation across studies attributable to between-study heterogeneity; values >50% indicate substantial heterogeneity. τ² represents the estimate of between-study variance in the random-effects model.

Supplementary Figure 4. Meta-analysis of cohort studies assessing unhealthful plant-based dietary index (uPDI) and breast cancer risk: forest plot showing pooled hazard ratios for the highest versus lowest index category.

Study	logHR	SE(logHR)	Weight (%)	HR	95%-CI
Martínez CF et al, 2023. Moli-Sani Study, Italy	0.0862	0.0611	16.1	1.09	[0.97; 1.23]
Romanos-Nanclares A et al, 2020. SUN cohort, Spain	0.2700	0.2601	1.5	1.31	[0.79; 2.18]
Romanos-Nanclares A et al, 2021. NHS and NHSII, USA	0.0392	0.0321	27.7	1.04	[0.98; 1.11]
Shah S et al, 2022. E3N, France	0.1823	0.0531	18.8	1.20	[1.08; 1.33]
Shah S et al, 2025. EPIC, Europe	0.0198	0.0351	26.3	1.02	[0.95; 1.09]
Thompson AS et al, 2023. UK Biobank, UK	0.1484	0.0899	9.6	1.16	[0.97; 1.38]
**Common effect model**			**.**	**1.07**	**[1.03; 1.11]**
**Random effects model**			**100.0**	**1.09**	**[1.02; 1.16]**

Heterogeneity: $I^2 = 43.1\%$, $\tau^2 = 0.0027$, $p = 0.1179$

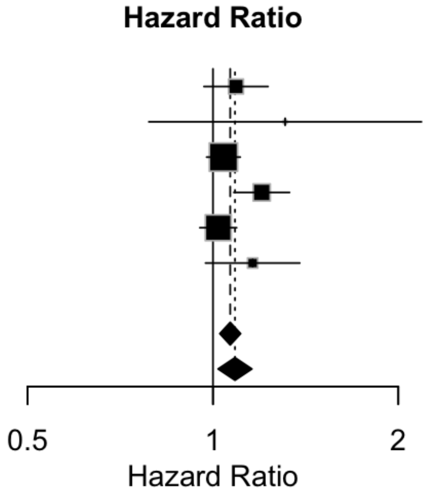

CI: confidence interval, HR: hazard ratio, SE: standard error, UK: United Kingdom, USA: United States of America. Study names: E3N: Étude Épidémiologique auprès de femmes de la Mutuelle Générale de l'Éducation Nationale, EPIC: European Prospective Investigation into Cancer and Nutrition, NHS: Nurses' Health Study. I^2 represents the percentage of total variation across studies attributable to between-study heterogeneity; values >50% indicate substantial heterogeneity. τ^2 represents the estimate of between-study variance in the random-effects model.

Supplementary Figure 5. Meta-analysis of cohort studies assessing plant-based/pro-vegetarian dietary index (PDI/PVG) and colorectal cancer risk: forest plot showing pooled hazard ratios for the highest versus lowest index category.

Study	logHR	SE(logHR)	Weight (%)	HR	95%-CI
Kim J et al, 2022. Multiethnic Cohort Study, USA (women)	-0.0101	0.0719	26.3	0.99	[0.86; 1.14]
Kim J et al, 2022. Multiethnic Cohort Study, USA (men)	-0.2744	0.0666	27.9	0.76	[0.67; 0.87]
Liu F et al, 2023. UK Biobank, UK	-0.1393	0.0641	28.6	0.87	[0.77; 0.99]
Yuan F et al, 2025. SCCS, USA	-0.1393	0.1106	17.1	0.87	[0.70; 1.08]
**Common effect model**			**.**	**0.86**	**[0.80; 0.93]**
**Random effects model**			**100.0**	**0.87**	**[0.77; 0.97]**

Heterogeneity: $I^2 = 58.9\%$, $\tau^2 = 0.0080$, $p = 0.0628$

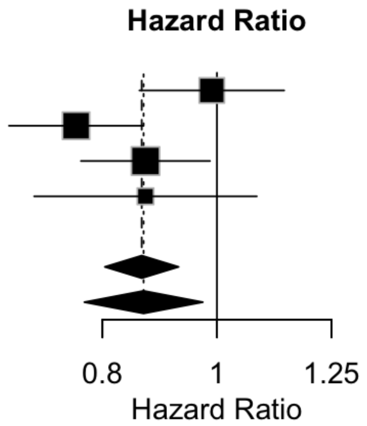

CI: confidence interval, HR: hazard ratio, SE: standard error, UK: United Kingdom, USA: United States of America. Study name: SCCS: Southern Community Cohort Study. I^2 represents the percentage of total variation across studies attributable to between-study heterogeneity; values >50% indicate substantial heterogeneity. τ^2 represents the estimate of between-study variance in the random-effects model. PDI and PVG were considered equivalent exposures due to their comparable construct and scoring.

Supplementary Figure 6. Meta-analysis of cohort studies assessing healthful plant-based dietary index (hPDI) and colorectal cancer risk: forest plot showing pooled hazard ratios for the highest versus lowest index category.

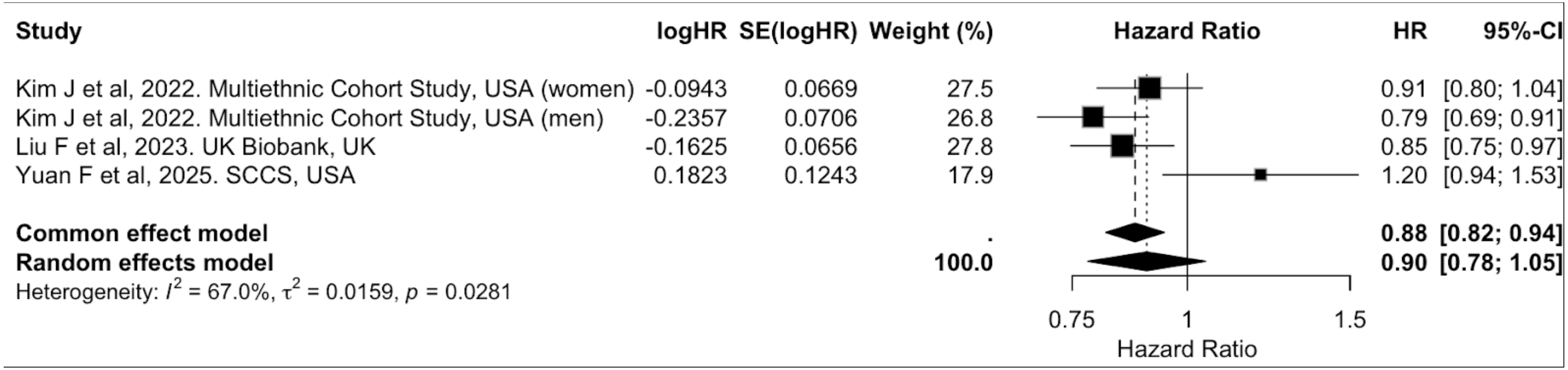

CI: confidence interval, HR: hazard ratio, SE: standard error, UK: United Kingdom, USA: United States of America. Study name: SCCS: Southern Community Cohort Study. I^2 represents the percentage of total variation across studies attributable to between-study heterogeneity; values >50% indicate substantial heterogeneity. τ^2 represents the estimate of between-study variance in the random-effects model.

Supplementary Figure 7. Meta-analysis of cohort studies assessing unhealthful plant-based dietary index (uPDI) and colorectal cancer risk: forest plot showing pooled hazard ratios for the highest versus lowest index category.

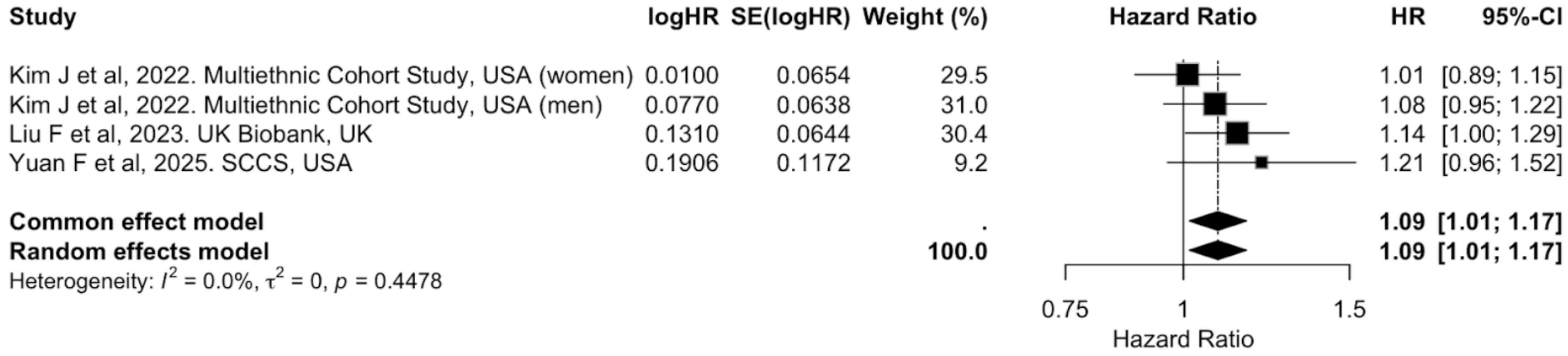

CI: confidence interval, HR: hazard ratio, SE: standard error, UK: United Kingdom, USA: United States of America. Study name: SCCS: Southern Community Cohort Study. I^2 represents the percentage of total variation across studies attributable to between-study heterogeneity; values >50% indicate substantial heterogeneity. τ^2 represents the estimate of between-study variance in the random-effects model.

Supplementary Figure 8. Meta-analysis of cohort studies assessing healthful plant-based dietary index (hPDI) and prostate cancer risk: forest plot showing pooled hazard ratios for the highest versus lowest index category.

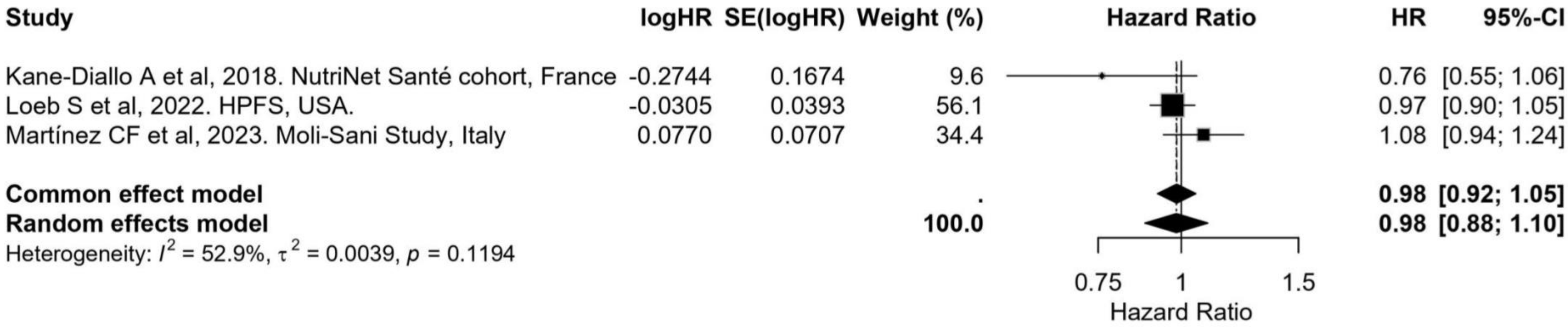

CI: confidence interval, HR: hazard ratio, SE: standard error, USA: United States of America. Study name: HPFS: Health Professionals Follow-up Study. I^2 represents the percentage of total variation across studies attributable to between-study heterogeneity; values >50% indicate substantial heterogeneity. τ^2 represents the estimate of between-study variance in the random-effects model. PDI and PVG were considered equivalent exposures due to their comparable construct and scoring.

Supplementary Figure 9. Meta-analysis of cohort studies assessing plant-based/pro-vegetarian dietary index (PDI/PVG) and prostate cancer risk: forest plot showing pooled hazard ratios for the highest versus lowest index category.

Study	logHR	SE(logHR)	Weight (%)	HR	95%-CI
Loeb S et al, 2022. HPFS, USA.	-0.0513	0.0406	54.0	0.95	[0.88; 1.03]
Martínez CF et al, 2023. Moli-Sani Study, Italy	0.0677	0.0686	21.9	1.07	[0.94; 1.22]
Thompson AS et al, 2023. UK Biobank, UK	-0.0202	0.0651	24.1	0.98	[0.86; 1.11]
**Common effect model**			.	**0.98**	**[0.92; 1.04]**
**Random effects model**			**100.0**	**0.98**	**[0.92; 1.05]**

Heterogeneity: I^2 = 10.2%, τ^2 = 0.0004, p = 0.3283

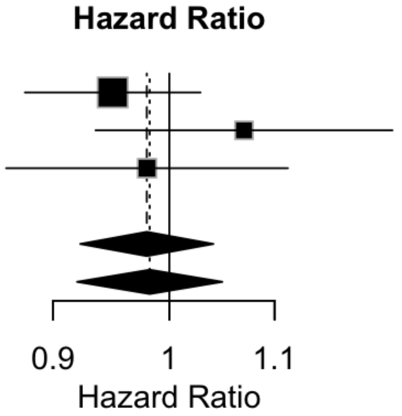

CI: confidence interval, HR: hazard ratio, SE: standard error, UK: United Kingdom, USA: United States of America. Study name: HPFS: Health Professionals Follow-up Study. I^2 represents the percentage of total variation across studies attributable to between-study heterogeneity; values >50% indicate substantial heterogeneity. τ^2 represents the estimate of between-study variance in the random-effects model.
